# Non-merohedral twinning: from minerals to proteins

**DOI:** 10.1107/S2059798319010179

**Published:** 2019-11-19

**Authors:** Madhumati Sevvana, Michael Ruf, Isabel Usón, George M. Sheldrick, Regine Herbst-Irmer

**Affiliations:** a Department of Biological Sciences, Purdue University, West Lafayette, IN 47907, USA; b Bruker Nano Inc., 5465 East Cheryl Parkway, Madison, WI 53711, USA; c Structural Biology, IBMB–CSIC, Baldiri Reixach 13-15, 08028 Barcelona, Spain; d ICREA, Passeig de Lluís Companys 23, 08010 Barcelona, Spain; e Department of Inorganic Chemistry, University of Göttingen, Tammannstrasse 4, 37077 Göttingen, Germany

**Keywords:** non-merohedral twinning, twinned structure solution, twinned structure refinement

## Abstract

In contrast to twinning by merohedry, the reciprocal lattices of the different domains of non-merohedral twins do not overlap exactly. This leads to three kinds of reflections: reflections with no overlap, reflections with an exact overlap and reflections with a partial overlap of a reflection from a second domain. This complicates the unit-cell determination, indexing, data integration and scaling of X-ray diffraction data. However, with hindsight it is possible to detwin the data because there are reflections that are not affected by the twinning. In this article, the successful solution and refinement of one mineral, one organometallic and two protein non-merohedral twins using a common strategy are described. The unit-cell constants and the orientation matrices were determined by the program *CELL_NOW*. The data were then integrated with *SAINT*. *TWINABS* was used for scaling, empirical absorption corrections and the generation of two different data files, one with detwinned data for structure solution and refinement and a second one for (usually more accurate) structure refinement against total integrated intensities. The structures were solved by experimental phasing using *SHELXT* for the first two structures and *SHELXC*/*D*/*E* for the two protein structures; all models were refined with *SHELXL*.

## Abstract 2

Examples are presented of successful data-processing, phasing and refinement strategies for non-merohedral twins, covering the range from minerals to proteins.

## 1. Introduction   

Twins are defined as regular aggregates consisting of individual crystals of the same species joined together in some definite mutual orientation (Giacovazzo, 2002 ▸). Therefore, twins may be defined by a symmetry operator that transforms one orientation into another, the so-called twin law, and by the fractional contribution *k*_*i*_ of each component. In reciprocal space, the twin law describes the symmetry operator that transforms the *h*_1_ *k*_1_ *l*_1_ indices of one domain into the indices *h*_2_ *k*_2_ *l*_2_ of a second domain.

Twins can be classified depending on the twin law (Herbst-Irmer, 2016 ▸; Herbst-Irmer & Sheldrick, 1998 ▸; Parsons, 2003 ▸; Yeates, 1997 ▸; Dauter, 2003 ▸; Banumathi *et al.*, 2004 ▸; Luo & Dauter, 2016 ▸). For merohedral and pseudo-merohedral twins the reciprocal lattices of the different domains overlap (nearly) exactly. Therefore, the intensities of reflection *h*_1_ *k*_1_ *l*_1_ of domain 1 and the twin-related reflection *h*_2_ *k*_2_ *l*_2_ of domain 2 sum up to a single observed intensity. This complicates the space-group determination and structure solution. However, after having solved the structure, refinement can be performed against these summed intensities and the fractional contribution *k*_*i*_ of each component can be refined. For pure merohedral twins of macromolecules, this has been automated in the programs *REFMAC* (Murshudov *et al.*, 2011 ▸) and *phenix.refine* (Adams *et al.*, 2010 ▸) and is widely used with good results, although sometimes just to lower the *R* factor of crystals that are not actually twinned. However, twinning should only be invoked when there is independent evidence apart from a lower *R* factor.

**Figure 1:**
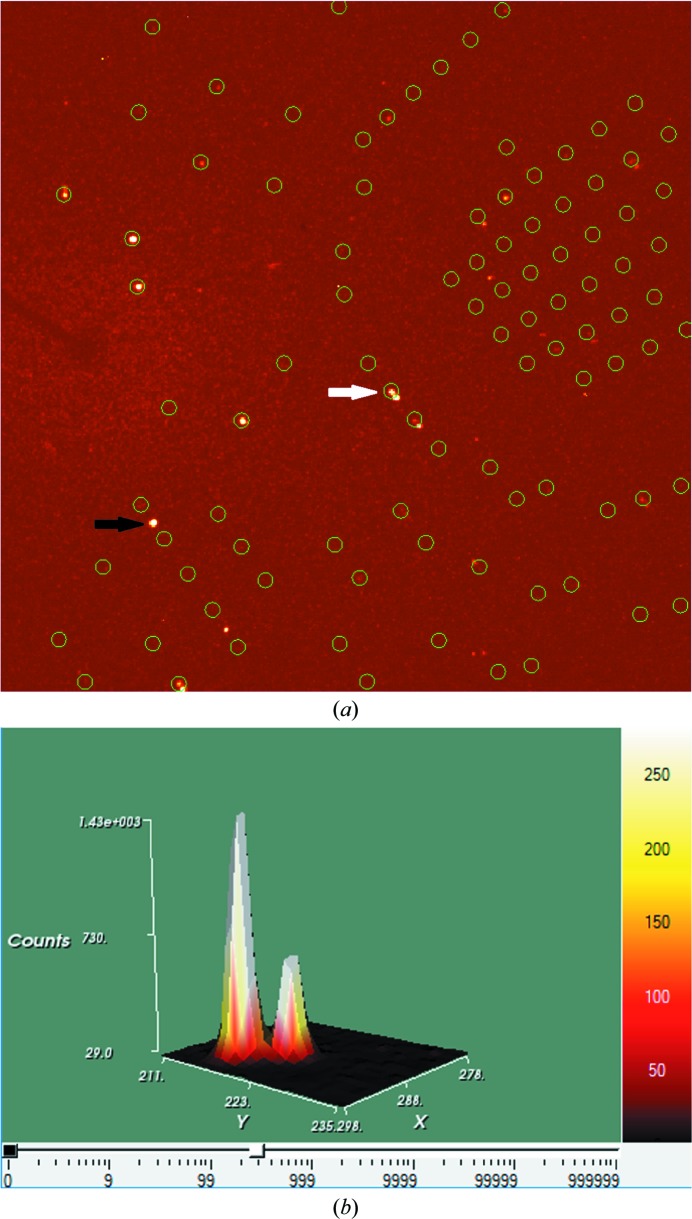
Diffraction patterns in *APEX* (Bruker’s crystallography software suite; Bruker, 2018 ▸) (*a*) indicating unindexed reflections (black arrow) and split reflections (white arrow) and (*b*) showing a split reflection profile

The twin law for non-merohedral twins does not belong to the crystal class or to the metric symmetry of the lattice. The different reciprocal lattices may not overlap exactly and not every reflection has contributions from all twin domains. Therefore, under normal circumstances this kind of twinning can be spotted during data collection. Quite often, autoindexing (automatic cell-determination) programs that were designed for single crystals fail or do not routinely handle multiple lattices to obtain the unit-cell parameters. Split reflection profiles can be observed and it may not be possible to index all reflections (see Fig. 1 ▸).

**Figure 2:**
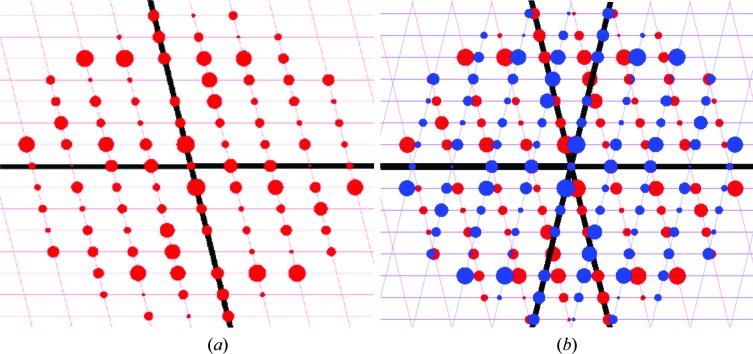
Reciprocal-space plot of the *k* = 2 layer of a monoclinic structure (*a*) and the overlay of this plot with a rotated plot simulating non-merohedral twinning (*b*).

To index reflections for a non-merohedral twin, more than one orientation matrix is required. Therefore, the autoindexing program must take into account that only a certain fraction of the reflections can be indexed as a single domain. After indexing the reflections from all of the domains, the data-integration program must be able to use all of the orientation matrices to obtain the intensities of reflections from the individual components (a simple strategy would be to integrate each component separately with its respective orientation matrix). This leads to three kinds of reflections: reflections with no overlap, reflections with an exact overlap and reflections with a partial overlap from further domains [see Fig. 2 ▸(*b*)]. The non-overlapped reflections are not affected by twinning. Both the non-overlapped and the exactly overlapped reflections can be used in model refinement. They determine the fractional contributions of the twin domains. For the partially overlapped reflections, the degree of overlap is unknown and therefore only a fraction of the reflections from the second domain can be integrated, so one option might be to omit reflections involving other domains. A second and much better strategy is to simultaneously integrate the reflections using orientation matrices from all of the components. Here, the overall intensity of every reflection is integrated, giving rise to two kinds of reflections: non-overlapped and overlapped reflections, which are also called single and composite reflections, respectively.

Standard scaling and absorption-correction programs cannot be used under these circumstances because special treatment is needed for composite reflections. Most of these challenges have been solved for twinned data from small-molecule crystals. Programs such as *DIRAX* (Duisenberg, 1992 ▸), *GEMINI* (Sparks, 2000 ▸), *CELL_NOW* (Sheldrick, 2008 ▸), *CrysAlis^Pro^* (Rigaku, 2015 ▸) and *MOSFLM* (Battye *et al.*, 2011 ▸) can index a diffraction pattern with more than one orientation matrix. The programs *SAINT* (Bruker, 2017 ▸), *EVAL*15 (Schreurs *et al.*, 2010 ▸), *X-Area* (Stoe & Cie, 2017 ▸) and *CrysAlis^Pro^* (Rigaku, 2015 ▸) can integrate with more than one orientation matrix simultaneously. Here, we describe the successful treatment of non-merohedral twins using the programs *CELL_NOW*, *SAINT* and *TWINABS* (Sheldrick, 2012 ▸), where *TWINABS* is a special version of *SADABS* (Krause *et al.*, 2015 ▸) that is used for scaling and absorption correction of data from non-merohedrally twinned crystals. Example structures of a mineral and an organo­metallic small molecule as well as two test protein structures will be discussed.

## 2. General strategy   

### 2.1. Cell determination   

The program *CELL_NOW* tries to find sets of equally spaced parallel reciprocal-lattice planes that pass close to as many reflections as possible. Each set of planes corresponds to a potential unit-cell vector perpendicular to the planes with a length given by the reciprocal of the inter-planar separation. Combinations of three such vectors form potential unit cells that are ranked by a figure of merit that favours the smallest possible unit-cell volume, the highest possible metric symmetry and the largest number of indexed reflections, *i.e.* reflections that lie within 0.2 times the interplanar separation from all three sets of planes.

*CELL_NOW* rotates each potential cell in turn to locate further twin domains by iteratively checking only those reflections that were not indexed by the cell in question. The rotation matrix from the first orientation to the second corresponds to the twin law. Therefore, the orientation matrices and the twin law are determined in one step. An additional advantage is that even weaker domains can be indexed. The alternative procedure of separately indexing the unindexed reflections from scratch might fail if there are too few reflections from the weaker domain in the list of harvested reflections for indexing.

### 2.2. Integration   

**Figure 3:**
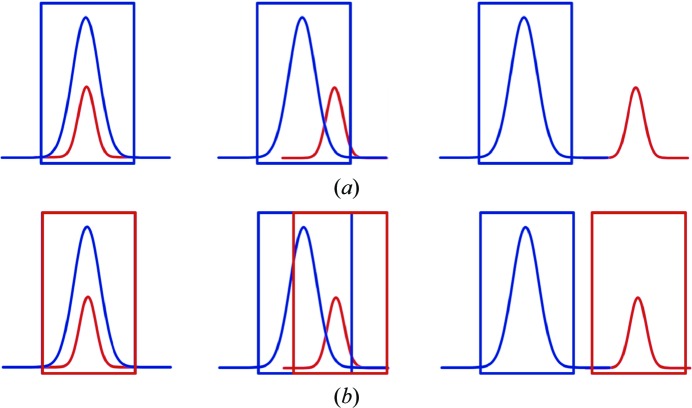
Schematic picture of reflections from two domains (blue and red) with different degrees of overlap. The rectangles represent the integration boxes. (*a*) Only one orientation matrix is used; (*b*) both orientation matrices are used.

In *SAINT*, a refineable integration box size is used. The intensity of non-overlapped reflections can be accurately determined when a single orientation matrix from one domain is used during data integration. However, the intensities determined for exactly overlapped reflections should be the sum of the intensities from all of the domains that contribute (see Fig. 3 ▸). The treatment of partially overlapped reflections is nontrivial, because the degree of overlap is unknown and differs from one reflection to the next. When using a single orientation matrix only, the measured intensity may be contaminated by contributions from other domains. However, in a simultaneous integration procedure with all of the orientation matrices from different domains it is possible to determine the overlap between the integration boxes of the reflections from different domains. The combined box size can then be used for integration, leading to the sum of all intensities from all of the domains. Using this procedure only two kinds of reflections remain: overlapped and non-overlapped reflections, which are also called single and composite reflections, respectively. For composite reflections, an additional column in the output raw data file specifies the domain numbers. Additionally, *SAINT* derives rough estimates of the individual intensities of the involved reflections by using the learnt reflection profile.

### 2.3. Absorption correction, scaling, merging and generation of datafiles   

The new raw datafile needs a special version of the scaling and absorption correction program *SADABS* (Krause *et al.*, 2015 ▸) called *TWINABS* (Sheldrick, 2012 ▸). The modelling of systematic errors such as absorption by the multi-scan method can be performed either for each domain separately by only using the non-overlapped reflections, or for reflections of several domains also considering overlapped reflections. *TWINABS* can detwin the data by using the rough overlap estimates from *SAINT* and refining these estimates using symmetry-related reflections. Symmetry-related non-overlapped reflections can only be merged if they belong to the same domain. For overlapped reflections, the ratios of the contributions from different domains need to be constant (for details, see the supporting information). In order to increase the number of unique data, reflections of all domains are used by default. Only if one or more domains are much weaker than the others does the program suggest using only single and composite reflections involving at least one of the stronger domains. This HKLF 4-format file with detwinned and merged data can be used in the same way as a standard HKLF 4 datafile from an untwinned single crystal for structure solution and refinement. Additionally, *TWINABS* produces a datafile containing summed intensities and the information about overlap in HKLF 5 format (further details of this format are given in the supporting information). The default option here is to use only reflections that contribute to the first domain.

All possible refinement programs can be used for the refinement against the HKLF 4 detwinned data. However, for small molecules the HKLF 5-format file containing the summed intensities with information about overlap and twin domains is often superior, *e.g.* example structure 4 in Herbst-Irmer (2016 ▸). In such cases refinement programs that are capable of handling this file can be used, for example *SHELXL* (Sheldrick, 2015*a* ▸), *OLEX*2 (Bourhis *et al.*, 2015 ▸) and *CRYSTALS* (Betteridge *et al.*, 2003 ▸).

## 3. Examples   

### 3.1. The mineral chromite   

**Figure 4:**
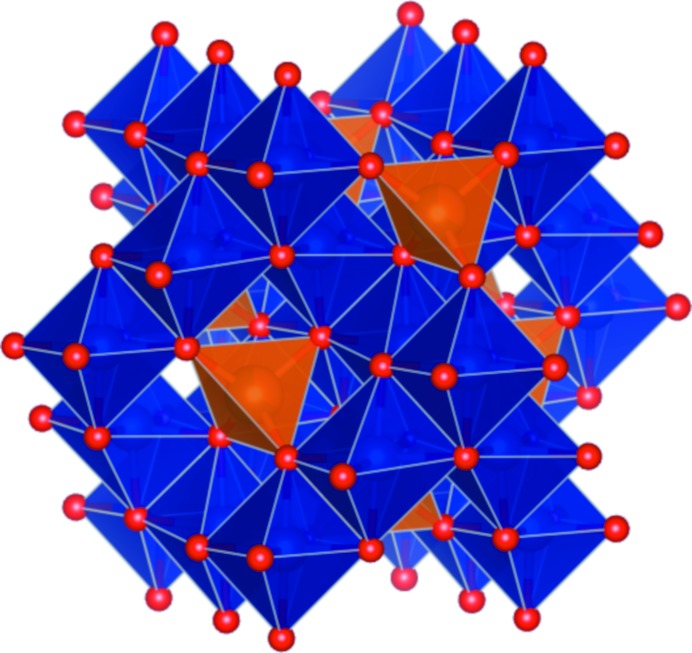
Structure of chromite with the Fe^2+^ tetrahedron in orange and the Cr^3+^ octahedron in blue, produced with *VESTA* v.3.4.6 (Momma & Izumi, 2011 ▸).

**Figure 5:**
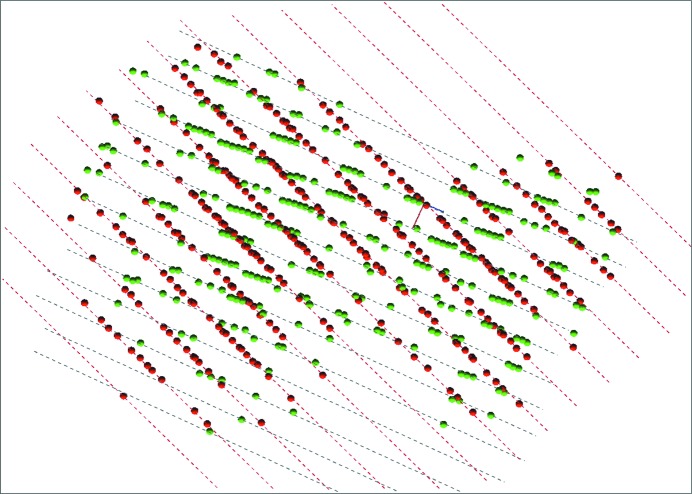
*RLATT* plot showing both orientations for chromite.

The mineral chromite, an iron chromium oxide FeCr_2_O_4_, crystallizes in the cubic space group *Fd* 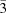 *m* (see Fig. 4 ▸). Iron can be substituted by magnesium in variable amounts (Lenaz *et al.*, 2004 ▸). A data set from a twinned crystal was collected using a Bruker D8 Quest at Mo *K*α wavelength at 292 K. Two domains could easily be identified using the graphical viewer *RLATT* (Bruker, 2016 ▸; see Fig. 5 ▸). *CELL_NOW* found a hexagonal cell with *a* = *b* = 5.88, *c* = 14.41 Å when the default settings were used (for details, see the supporting information). The systematic absences for the obverse setting could not be identified by the program because 11.5% of the indexed reflections are outliers. This obverse cell can be transformed to the true cubic *F*-centred cell. On restricting the vector search for cell edges between 8 and 9 Å, the correct *F*-centred cubic cell was identified, indexing 58.6% of the harvested reflections. 0.5% of the reflections violate the systematic absences for the *F*-centring. Rotating this cell by 180° around −2 −1 1 led to a second orientation matrix that indexed 94.6% of the hitherto unindexed reflections (for details, see the supporting information).

Both orientation matrices were used in *SAINT* for integration, which produced the raw datafile with information about the domain overlap for individual reflections. *TWINABS* distinguished three types of reflections: singles from domain 1, singles from domain 2 and composite reflections (see Table 1 ▸ and the supporting information). Parameter refinement was applied separately for both domains using only single reflections. The detwinning procedure estimated a twin fraction of 0.574 for the major domain, with *R*_int_ values of 0.0489 for both domains and 0.0439 using only data from the major domain. The merged data files consisted of only 69 reflections. *SHELXT* (Sheldrick, 2015*b* ▸) could solve the structure immediately using the detwinned data. The refinement can be performed against either the detwinned HKLF 4 data set or the HKLF 5 data set consisting of reflections from domain 1, domain 2 or both domains. The results from refinements using different HKLF 5 files are comparable. Domain 2 was weaker than the other domain, with slightly worse figures of merit.

### 3.2. Organometallic example   

**Figure 6:**
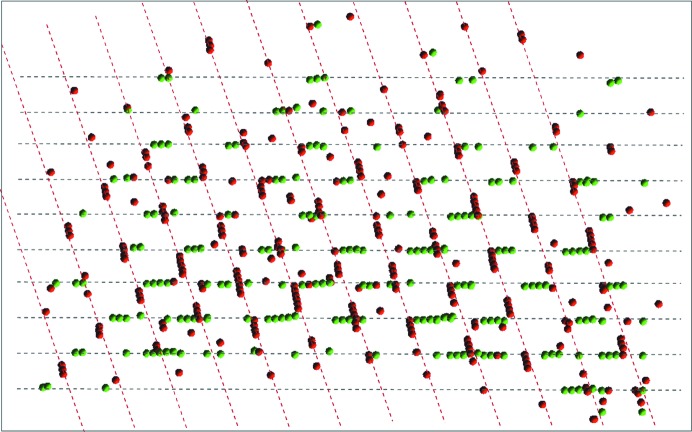
*RLATT* plot showing both orientations for Cp*_2_MeZrOTiMe_2_Cp*.

The compound Cp*_2_MeZrOTiMe_2_Cp* (where Cp* is pentamethylcyclopentyl) crystallizes as a non-merohedral twin (Gurubasavaraj *et al.*, 2007 ▸). A data set was collected at 100 (2) K using a Bruker SMART APEX II diffractometer with a D8 goniometer (graphite-monochromated Mo *K*α radiation). Indexing with automatic single-crystal cell-determination programs failed. Two domains could easily be identified using *RLATT* (see Fig. 6 ▸). *CELL_NOW* produced an extensive list of 172 possible cells with different cell volumes but with very similar percentages of indexed reflections (for details, see the supporting information and Table 2 ▸). The first cell indexed 54.6% of the reflections with an *I*-centred monoclinic cell. After a rotation of 180° about the 0 1 1 reciprocal axis, 69.2% of the as-yet unindexed reflections could be indexed with a second orientation matrix. No further meaningful orientation matrices were found. Therefore, an initial cell with a slightly higher percentage of indexed reflections (Cell 4 in Table 2 ▸) and a doubled cell volume for a primitive monoclinic cell was chosen. After rotating by 180° about the 0 −1 2 reciprocal axis, all of the remaining reflections could be indexed.

These two orientations were used in *SAINT* for integration. *TWINABS* indicated that the two domains are rather similar in size (see Table 3 ▸). The systematic absences are consistent with space group *P*2_1_/*c* (see the supporting information), but *SHELXT* correctly identified *Pc* as the true space group. By default, *TWINABS* merges Friedel opposites, but this option can be changed for non-centrosymmetric space groups. In principle, for non-centrosymmetric structures additional twinning by inversion is possible. There is an additional option in *TWINABS* to generate an HKLF 5 file using four domains: the major domain 1, the minor domain 2, the inverse of domain 1 and the inverse of domain 2. For this data set, the fractional contributions *k*_*i*_ refined to *k*_2_ = 0.45 (3), *k*_3_ = 0.50 (3) and *k*_4_ = 0.02 (3), where *k*_1_ = 1 − (*k*_2_ + *k*_3_ + *k*_4_). These values indicated that the absolute structure is wrong for domain 1 but correct for domain 2. Therefore, the atomic coordinates had to be inverted in *SHELXL* and additionally the indices of the reflections of the second domain had to be inverted in *TWINABS*. The final results with this option are listed in Table 3 ▸. The HKLF 4 and HKLF 5 files gave similar results. However, judging from the *R* value after dispersion correction and merging, which has the same number of reflections for all refinements, the data set using complete data from both domains produces better results. This can be explained by the fact that both domains are similar in size and both are well centred in the beam (see the normalized scale-factor plot in the supporting information).

**Figure 7:**
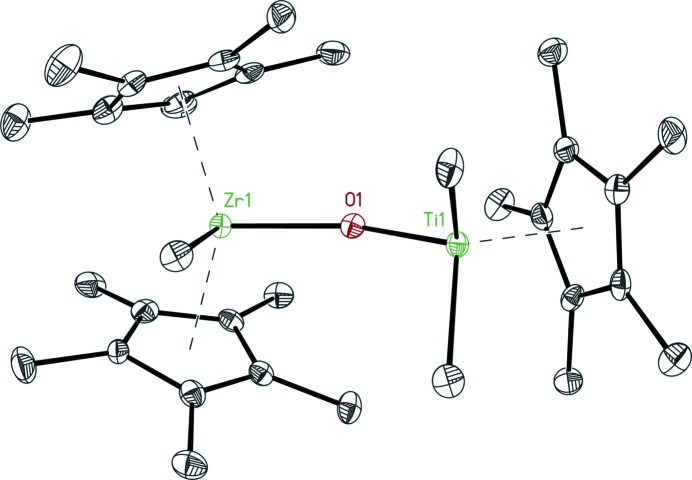
Structure of one of the two molecules of Cp*_2_MeZrOTiMe_2_Cp*.

There are two molecules in the asymmetric unit in space group *Pc* (see Fig. 7 ▸). There is no inversion centre or 2_1_ axis between the two molecules. However, there is a pseudo-2_1_ axis relating the Zr atom of molecule 2 to the Ti atom of molecule 1 and vice versa (for details, see the supporting information). Additionally, there is a pseudo-translation between the two Zr atoms and the two Ti atoms. They are related by *x* + 0.5, *y* + 0.5, *z* + 0.25, which would lead to *I*-centring if the *c* cell axis were to be halved. This corresponds to the smaller cell proposed by *CELL_NOW* (see Table 2 ▸). It also explains why the true cell indexes only 60% of the reflections compared with 54% for this smaller cell, which has a four times smaller primitive volume. Owing to the pseudo-translation, there are many weak reflections that will not be found in the list of reflections from the peak search.

## 4. Twinned protein crystals   

The methods described above have successfully been used for twinned small molecules for many years, and *SHELXD* and *SHELXE* have also been used to assist in the SAD phasing of merohedrally twinned macromolecules (Dauter, 2003 ▸; Rudolph *et al.*, 2003 ▸). To show that the procedures described in this paper are also valid for macromolecular structures, we grew non-merohedrally twinned crystals of two benchmark protein structures: cubic insulin and glucose isomerase (Sevvana, 2006 ▸; Fig. 11, right).

Both data sets were collected at 100 K with ω scans using a Bruker rotating-anode generator at Cu *K*α wavelength equipped with Osmic focusing mirrors and a Bruker SMART6000 4K detector. The data were collected in low-, medium- and high-resolution passes at detector distances of 10 or 18 cm in thin-slice mode to minimize artificial overlap of the spots because of detector geometry. A minimum of three runs for each of the low-, medium- and high-resolution passes were collected at different φ angles to obtain complete and multiple observations of data in order to maximize the weak anomalous signal from sulfur (in the case of cubic insulin) and manganese (in the case of glucose isomerase) at the Cu *K*α wavelength. It was important to collect data as precisely as possible, avoiding ice rings *etc.*, so that the only problems that were encountered during data processing were caused by twinning.

**Figure 8:**
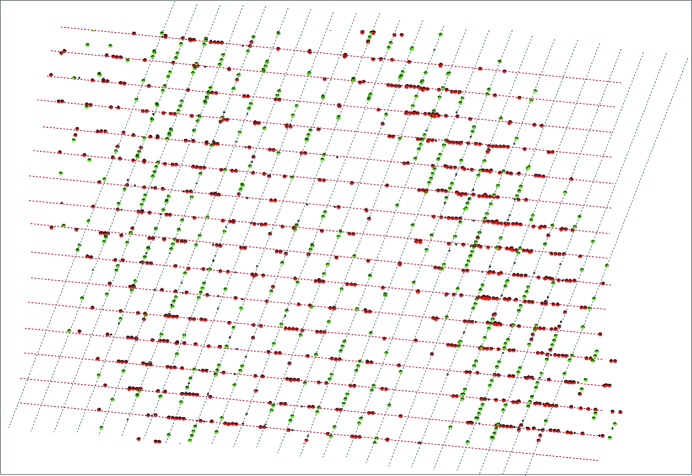
*RLATT* plot showing the two orientations of insulin.

The complications of data collection using these twinned protein crystals were similar to the small-molecule examples. Automatic cell determination failed, but both *RLATT* (see Fig. 8 ▸) and *CELL_NOW* (see the supporting information) clearly identified two domains in the case of insulin and three domains for glucose isomerase (see Fig. 10), and their orientation matrices were used in *SAINT* in the same way as for the small molecules. *TWINABS* produced detwinned HKLF 4 data and several HKLF 5 data sets. Both substructures were solved using dual-space recycling methods in *SHELXD* (Schneider & Sheldrick, 2002 ▸). The normalized difference structure factors were calculated using *XPREP* from the HKLF 4 file prepared by *TWINABS*. Density modification and autotracing were carried out using *SHELXE* (Usón *et al.*, 2007 ▸; Usón & Sheldrick, 2018 ▸). *PDB*2*INS* (Lübben & Sheldrick, 2019 ▸) was used to convert the .pdb file to a SHELX.ins file. Both the insulin and glucose isomerase models were refined using *SHELXL* by alternating with model building in real space using *Coot* (Emsley *et al.*, 2010 ▸).

Although refinement of the models against the HKLF 5 files could produce better results, one of the challenges is to annotate the *R*_free_ reflections in this file format. It should be ensured that twin-related reflections are either both in the work set or both in the free set. For (pseudo)-merohedral twins this can be achieved by assigning them in thin shells instead of randomly in *XPREP* (Sheldrick, 2015*c* ▸). Because of the exact overlap of the different reciprocal lattices, twin-related reflections have the same θ value. In the case of non-merohedral twins the θ values could differ slightly. Therefore, one of the twin-related reflections could be in the θ shell for the free reflections, while the other is in the shell of the work reflections. Depending on the degree of overlap, it might be possible to derive a *R*_free_ set by successively adding these work reflections into the free set. In our example structures we ended with ∼90% of the reflections in the *R*_free_ set, even when we started with just one reflection in the first *R*_free_ set (for details, see the supporting information). The residual 10% could also not be used as an *R*_free_ set because they do not represent the whole data set. If one takes only single reflections as *R*_free_ reflections, it is questionable whether these reflections are a random representative of the whole data set. For our insulin data set, only 10% of the data were single. Additionally, the standard *R*_free_ procedure in *SHELXL* is not possible for the HKLF 5 format, because the information about overlap and twin domains is given in the same column as the identification of *R*_free_ reflections. This could be solved by either using the detwinned data or separating the work data and the free data into two separate files. However, if we assume that the detwinning works perfectly, it is no longer necessary to take care of twin-related reflections and the usual procedures for selecting the *R*_free_ reflections can be used for the detwinned (HKLF 4) data.

It is also known that all *R* values of structures from twinned crystals are artificially too low (Murshudov, 2011 ▸). This is also observed here for the refinements against the different HKLF 5 data sets, which show lower *R* values than refinements against the detwinned data. The latter values seem to be more realistic.

In order to judge whether a model derived by refinement against the HKLF 5 data is superior to the model derived from the detwinned data, the *R* values of these models against the detwinned data were calculated by refining just the scale factor. This was inspired by the procedure of paired refinement developed by Diederichs and Karplus (Diederichs & Karplus, 2013 ▸; Karplus & Diederichs, 2012 ▸).

### 4.1. Cubic insulin   

Bovine insulin (Sigma; catalogue No. I5500) was dissolved in 0.02 *M* Na_2_HPO_4_ and 0.01 *M* Na_3_EDTA to a final concentration of 30 mg ml^−1^. Crystals were grown by the hanging-drop vapour-diffusion method at 20°C by equilibration against a reservoir consisting of 0.2 *M* Na_2_HPO_4_/Na_3_PO_4_ pH 10.0, 0.01 *M* Na EDTA. Cubic crystals grew in about 1 h, and most of the crystals were interpenetrant owing to the high concentration of protein (which was deliberate in order to encourage the growth of twinned crystals) [Fig. 11(*a*), right].

Cubic insulin crystallizes in space group *I*2_1_3, which belongs to the lower symmetry cubic Laue group. Therefore, there are two independent possibilities for indexing the reflections related by the matrix (0 1 0, 1 0 0, 0 0 −1). The integration of two sets of reflections with different indexing leads to artificial merohedral twinning. Therefore, one has to be careful when indexing two different domains. In our case, *CELL_NOW* indexed the two domains using alternative settings. However, this was easily identified in *TWINABS*. The program detwins the data using an iterative process minimizing the *R*_int_ value for symmetry-equivalent reflections. Here, *TWINABS* advised converting the indices of component 2 by applying the matrix (0 1 0, 1 0 0, 0 0 −1), decreasing *R*_int_ to 0.0347.

**Figure 9:**
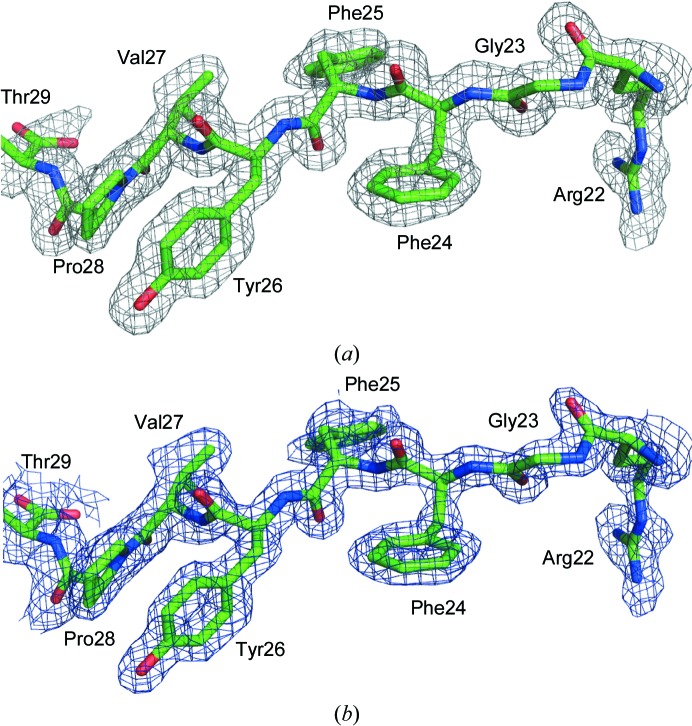
Part of the *SHELXE* map (*a*) and the final refined map (*b*) for cubic insulin contoured at 1σ.

The resulting detwinned data set extends to a maximum resolution of 1.55 Å. The structure contains 51 amino acids in two chains connected by three disulfide bonds. Both the higher symmetry space group and the three disulfide bonds make cubic insulin an ideal crystal for structure solution using in-house sulfur-SAD. To locate the anomalous scatterers the data were truncated to 1.9 Å resolution and *E*-values (normalized difference structure factors) were calculated in *XPREP*. Using these data, *SHELXD* (Sheldrick *et al.*, 2012 ▸) found the positions of three disulfide bridges (see the supporting information). Density modification and autotracing modules in a beta version of *SHELXE* could trace two chains. Sequence information was read from a file in FASTA format, and probing γ positions and side-chain shape along with the sulfur sites in the substructure was used to dock the polyalanine trace into the sequence after the last main-chain tracing cycle. Side chains were then built and refined. The total overhead for side-chain tracing was 0.2 s and the CC (Fujinaga & Read, 1987 ▸) from the trace against the normalized observed amplitudes increased from 40.0% (for a polyalanine trace as in previous versions of *SHELXE*) to 58.2% (for almost complete side chains). 86% of the side chains were traced with a largest side-chain difference within 1.5 Å and 8% with a greater difference, while 6% were missing or wrong (see Fig. 9 ▸). The missing side chains and the water structure were built in *Coot* (Emsley *et al.*, 2010 ▸) and the model was refined using *SHELXL*. The results of all refinements of this final model against the different datafiles are summarized in Table 4 ▸.

Both domains were well centred in the beam (for details see Fig. 11) and the quality of the data from the different domains is very similar. Both the HKLF 4 and HKLF 5 data yield very similar models, but the *R* values for the HKLF 5 refinement in this and other examples appear to be artificially low. Since it also can be problematic to obtain a suitable set of reflections for the free-*R* test in the HKLF 5 case, it is better to use the HKLF 4 data for *R*_free_.

### 4.2. Glucose isomerase   

The active form of glucose isomerase consists of 385 amino acids with eight methionines, a magnesium ion and a manganese ion at the active site. Glucose isomerase (Hampton Research; catalogue No. HR7-102) was dialysed against 5 m*M* Tris–HCl buffer pH 7.5, 10 m*M* MnCl_2_, 5 m*M* MgCl_2_ and then concentrated to a final concentration of 20 mg ml^−1^ and crystallized by the hanging-drop vapour-diffusion method by equilibration against a reservoir consisting of 0.05 m*M* Tris–HCl buffer pH 7.5, 0.1 *M* MnCl_2_, 14% MPD. The crystals grew in about two days. In contrast to the interpenetrant twinned crystals of bovine insulin, here it appears that three separate crystals grew in contact with each other [Fig. 11(*b*), right]. 25% MPD was used as a cryoprotectant and data were collected at 100 K with a detector distance of 18 cm because of the long cell axis.

**Figure 10:**
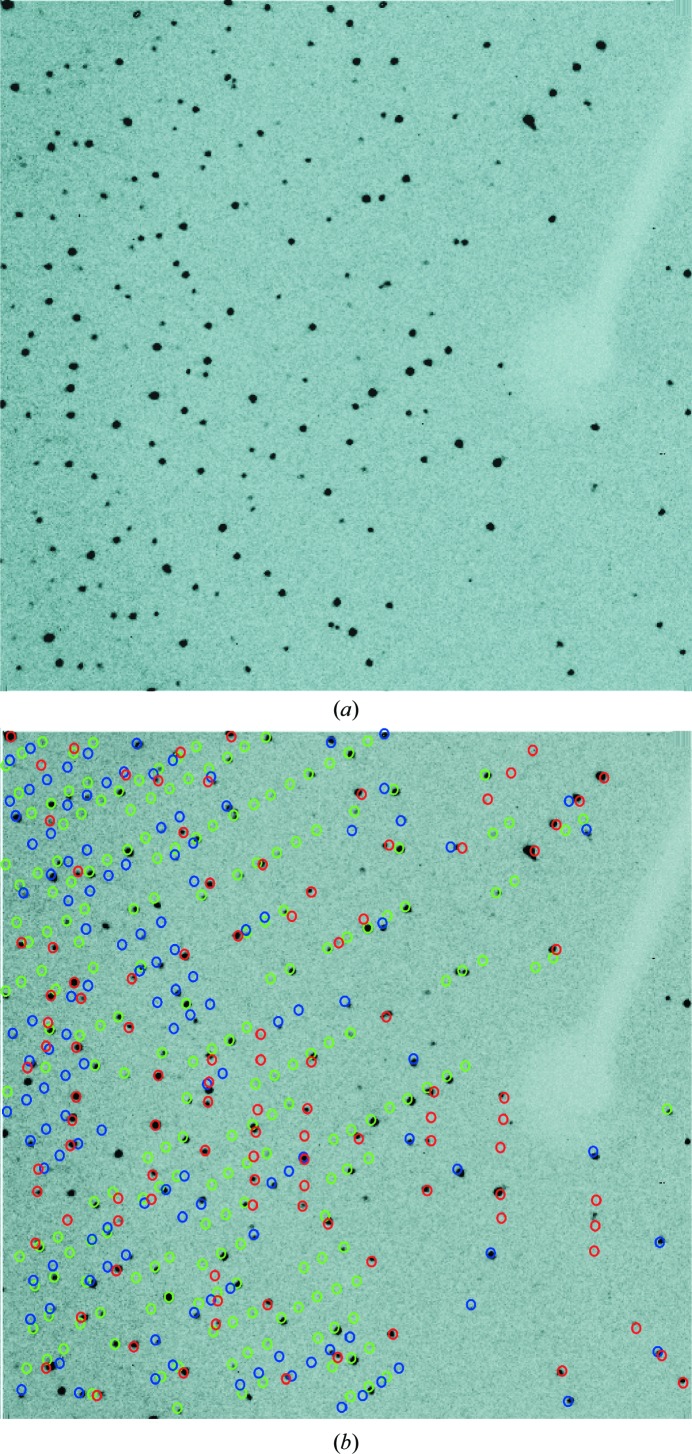
An example image of a glucose isomerase triplet. (*a*) An image taken at 2θ = 40° and a detector distance of 18 cm. (*b*) The indexed image using *CELL_NOW*. The first domain is coloured blue, the second domain is in green and the third domain is in red.

**Figure 11:**
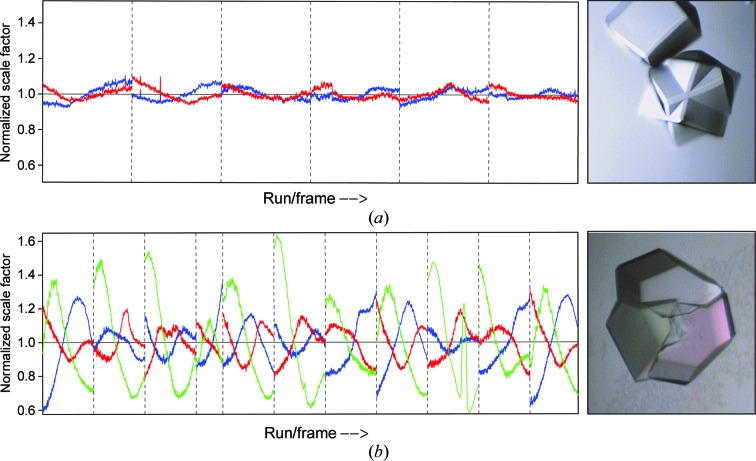
Normalized scale factor against run/frame number from *TWINABS* for (*a*) cubic insulin and (*b*) glucose isomerase; domain 1 is coloured blue, domain 2 is in red and domain 3 (only for the triple twin of glucose isomerase) is in green. The corresponding crystal pictures demonstrate the correlation between crystal growth and different centring in the beam.

**Figure 12:**
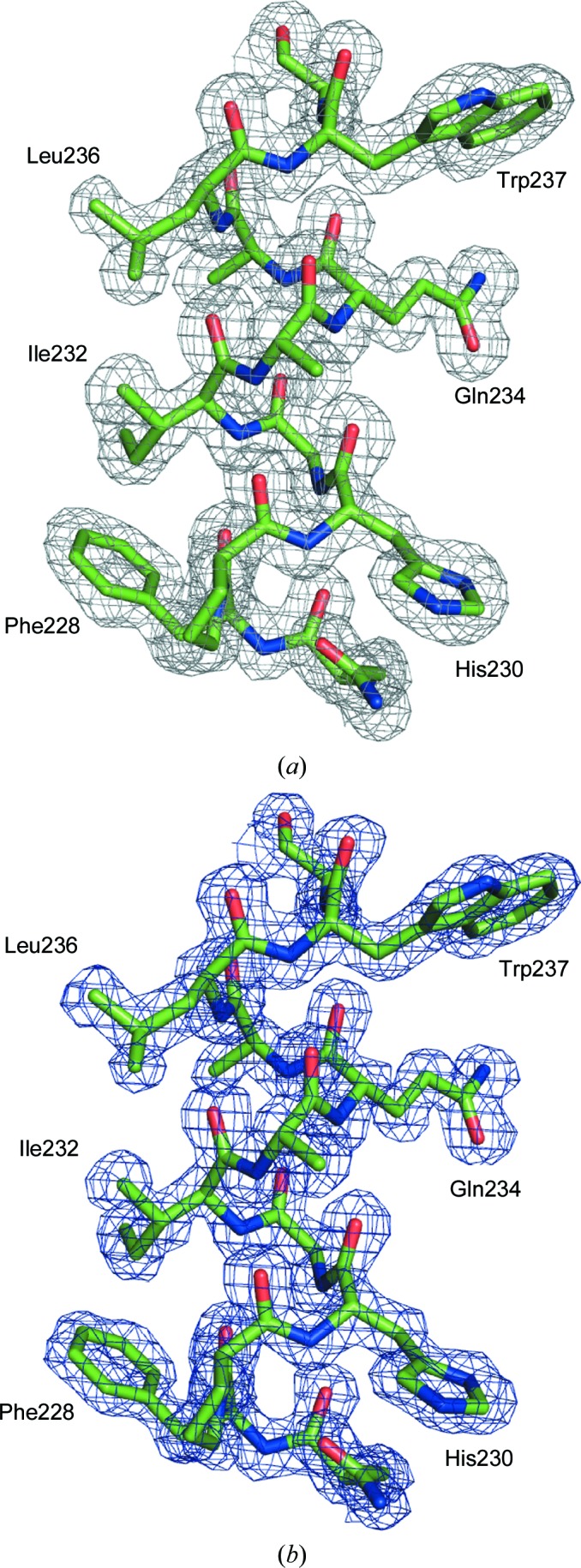
Part of the *SHELXE* map (*a*) and the final refined map (*b*) for glucose isomerase contoured at 1σ.

For glucose isomerase, *CELL_NOW* found three different orientation matrices (for details, see Fig. 10 ▸ and the supporting information) for data integration in *SAINT*. The scaling procedure in *TWINABS* indicated that the larger domains 1 and 2 with fractional contributions of 0.44 and 0.41 (see Table 5 ▸) were much better centred in the beam [Fig. 11 ▸(*b*)]. The detwinned data extended to a resolution of 1.6 Å and were truncated to 2.0 Å resolution for substructure solution of the two Mn sites in *SHELXD*. One site has a much lower peak height (see the supporting information), which was interpreted as a mixture of Mn and Mg. Density modification and autotracing of the inverted substructure in *SHELXE* identified 352 residues in ten chains. The total overhead for side-chain tracing was 2.3 s and the CC of the trace against the normalized observed amplitudes increased from 38.5% (for a polyalanine trace as in previous versions of *SHELXE*) to 50.0% (for almost complete side chains). 71.7% of the side chains were traced with a largest side-chain difference within 1.5 Å and 10.3% with a greater difference, while 17.8% were missing or wrong (see Fig. 12 ▸). The model was further improved by alternating refinement in *SHELXL* and model building in *Coot*. The second Mn site was partly occupied by Mg. The Mn and Mg atoms were constrained to have the same isotropic displacement parameter and *x*, *y*, *z* coordinates. The occupancy of the Mg atom refined to 0.64 (6). This is in accordance with the peak heights in the anomalous map. All atoms were refined isotropically with appropriate restraints. The addition of H atoms as well as anisotropic refinement increased the *R*_free_ value.

As in the case of insulin, all refinements against the different data sets are of similar quality. Again, the higher the multiplicity the better the models are. The difference between the HKLF 5 models and the model of the detwinned data is negligible, so there is no requirement for refinement against the HKLF 5 data.

The scale-factor plots in Fig. 11 ▸ show little variation with rotation angle for cubic insulin [Fig. 11 ▸(*a*)] because the two interpenetrating crystals have virtually the same centres, but for the cluster of three glucose isomerase crystals [Fig. 11 ▸(*b*)] there are substantial variations, especially for the smallest crystal 3 (green) that is furthest from the beam centre.

## 5. Conclusions   

The same procedures may be used for the treatment of non-merohedral twins in minerals, organometallic structures and proteins when the data are processed using the programs *CELL_NOW*, *SAINT* and *TWINABS*. *CELL_NOW* and *SAINT* are also incorporated into the Bruker *APEX*3 system. The resulting HKLF 4- and HKLF 5-format files can be used for structure solution and refinement with the *SHELX* and several other program systems. The detwinned HKLF 4 data are more widely applicable, but refinement against the composite reflections without detwinning using the HKLF 5 format may be slightly more accurate. If all domains are of similar quality and all of them are well centred in the beam, refinement against the HKLF 5 data should lead to the best results because the multiplicity is the highest. Quite often data from one domain might be of superior quality to those from other domains. In this case, only reflections with a contribution from that domain should be used for model refinement. However, in order to use *R*_free_ the HKLF 4 format may be required.

## Supplementary Material

PDB reference: insulin, 6or0

PDB reference: glucose isomerase, 6oqz

Crystal structure: contains datablock(s) chromite_4, chromite_5_1, chromite_5_2, chromite_5_12, zrti_4, zrti_5_1, zrti_5_2, zrti_5_12. DOI: 10.1107/S2059798319010179/rr5182sup1.cif

Supporting information including Supplementary Figures and Tables. DOI: 10.1107/S2059798319010179/rr5182sup2.pdf

Structure factors: contains datablock(s) chromite_4. DOI: 10.1107/S2059798319010179/rr5182chromite_4sup3.hkl

Structure factors: contains datablock(s) chromite_5_1. DOI: 10.1107/S2059798319010179/rr5182chromite_5_1sup4.hkl

Structure factors: contains datablock(s) chromite_5_2. DOI: 10.1107/S2059798319010179/rr5182chromite_5_2sup5.hkl

Structure factors: contains datablock(s) chromite_5_12. DOI: 10.1107/S2059798319010179/rr5182chromite_5_12sup6.hkl

Structure factors: contains datablock(s) zrti_4. DOI: 10.1107/S2059798319010179/rr5182zrti_4sup7.hkl

Structure factors: contains datablock(s) zrti_5_1. DOI: 10.1107/S2059798319010179/rr5182zrti_5_1sup8.hkl

Structure factors: contains datablock(s) zrti_5_2. DOI: 10.1107/S2059798319010179/rr5182zrti_5_2sup9.hkl

Structure factors: contains datablock(s) zrti_5_12. DOI: 10.1107/S2059798319010179/rr5182zrti_5_12sup10.hkl

Non-merohedral twinning: from minerals to proteins (insulin data set).: https://doi.org/10.18430/m3.irrmc.5325

Non-merohedral twinning: from minerals to proteins (isomerase data set).: https://doi.org/10.18430/m3.irrmc.5324

CCDC references: 1940918, 1940919, 1940920, 1940921, 1940922, 1940923, 1940924, 1940925

## Figures and Tables

**Table 1:** Data and refinement statistics for the mineral example

Domain	1	2	Both	Detwinned
*TWINABS*				
No. of data	675	659	45	—
No. of unique data	69	69	19	—
*I*/σ(*I*)	60.4	51.9	82.4	—
*R*_int_	0.0439	—	0.0489	—
*k*_*i*_	0.574	0.426	—	—
*SHELXL*				
Data used	60	60	138	60
Unique data used	60	60	60	60
Completeness (%)	97.4	97.4	97.4	97.4
No. of parameters	10	10	10	9
*R*1 [*I* > 2σ(*I*)]	0.0189	0.0271	0.0264	0.0161
*wR*2 (all data)	0.0521	0.0697	0.0680	0.0441
Bond precision (Cr—O) (Å)	0.0017	0.0030	0.0017	0.0015
*R*1 (after dispersion correction and merging)	0.0185	0.0269	0.0174	0.0166
*k*_2_	0.463 (10)	0.417 (11)	0.423 (5)	—

**Table 2:** Excerpt of *CELL_NOW* output: list of possible cells for Cp*_2_MeZrOTiMe_2_Cp*

	FOM	Indexed (%)	*a* (Å)	*b* (Å)	*c* (Å)	α (°)	β (°)	γ (°)	*V* (Å^3^)	Lattice type
1	1.000	54.6	8.676	15.514	11.578	89.93	94.47	89.87	1553.7	*I*
2	0.846	54.2	13.923	15.514	8.676	89.87	123.97	90.18	1554.2	*C*
3	0.723	60.0	23.245	30.889	8.676	90.06	94.46	90.05	6210.8	*C*?
**4**	**0.720**	**60.0**	**8.676**	**15.514**	**23.168**	**90.02**	**94.49**	**90.13**	**3108.8**	***P***
5	0.678	59.6	23.168	31.009	8.676	90.11	94.49	90.05	6213.8	*C*?
6	0.583	58.4	8.676	15.452	23.245	90.03	94.46	90.07	3106.8	*P*
7	0.540	55.6	8.676	10.399	10.434	96.13	111.98	111.85	776.5	*P*
8	0.535	56.0	8.676	10.399	10.782	66.27	63.70	68.15	775.8	*P*

**Table 3:** Data and refinement statistics for Cp*_2_MeZrOTiMe_2_Cp*

Domain	1	2	Both	Detwinned
*TWINABS*				
No. of data	30843	30852	4354	—
No. of unique data	5738	5739	1814	—
*I*/σ(*I*)	3.1	2.9	4.8	—
*R*_int_	0.0951	0.0992	0.0976	—
Fractional contribution	0.532	0.468		
*SHELXL*				
*R*1 [*I* > 2σ(*I*)]	0.0581	0.0588	0.0624	0.0481
Data used	11343	11325	24945	11172
Unique data used (Friedel pairs merged)	5596	5595	5596	5596
Completeness (%)	100	100	100	100
No. of parameters	686	686	686	685
*wR*2 (all data)	0.1260	0.1246	0.1314	0.1019
Bond precision C—C (Å)	0.0131	0.0136	0.0136	0.099
*R*1 (after dispersion correction and merging)	0.0527	0.0548	0.0456	0.0450
*k*_2_	0.4752 (19)	0.4640 (17)	0.4691 (10)	—

**Table 4:** Data and refinement statistics for cubic insulin Raw data have been deposited in the Integrated Resource for Reproducibility in Macromolecular Crystallography (Grabowski *et al.*, 2016 ▸; https://proteindiffraction.org) at https://doi.org/10.18430/m3.irrmc.5325.

Domain	1	2	Both	Detwinned
PDB code	6or0	6or0	6or0	6or0
Space group	*I*2_1_3	*I*2_1_3	*I*2_1_3	*I*2_1_3
*a* = *b* = *c* (Å)	78.03 (8)	78.03 (8)	78.03 (8)	78.03 (8)
Mosaicity (°)	0.33	0.33	0.33	0.33
Resolution (Å)	1.55	1.55	1.55	1.55
*TWINABS* data statistics				
No. of data	202583	202218	29318	—
No. of unique data	11532	11502	19152	—
*I*/σ(*I*)	9.4	7.5	10.2	—
*R*_int_	0.0336	0.0382	0.0347	—
*R*_r.i.m_ †	0.0345	0.0392	0.0352	—
Fractional contribution	0.581	0.419	—	—
Overall *B* factor from Wilson plot (Å^2^)	12.70	12.51	12.55	12.35
*SHELXL* refinement statistics				
*R*1 [*I* > 4σ(*I*)]	0.112	0.109	0.128	0.158
Data used	21453	21417	42151	11668
Unique data used	11560	11531	11663	11668
Completeness (%)	97.4	97.2	98.3	98.4
No. of parameters	3855	3855	3855	3854
*wR*2 (all data)	0.305	0.301	0.350	0.386
*R*1 (after dispersion correction and merging)	0.140	0.131	0.163	0.168
*k*_2_	0.428 (3)	0.414 (4)	0.420 (3)	—
*R*1(free) (all 588 data)	—	—	—	0.215
*R*1 (after dispersion correction and merging) against the detwinned data	0.173	0.174	0.164	—
Solvent content (%)	65	65	65	65
No. of non-H atoms				
Protein	395	395	395	395
Water	34	34	34	34
R.m.s.d., bonds (Å)	0.0137	0.0136	0.0212	0.0102
R.m.s.d., angles (°)	2.40	2.52	3.11	1.95
Average *B* factors (Å^2^)				
Main chain	16.67	16.76	16.67	16.35
Side chain and water	26.29	26.44	27.42	24.89
Ramachandran plot				
Most favoured (%)	97.83	95.65	97.83	97.83
Allowed (%)	2.17	4.35	2.17	2.17
Outliers (%)	0.0	0.0	0.0	0.0

† Calculated as [*N*/(*N* − 1)]^1/2^ × *R*_int_, where *N* is the data multiplicity.

**Table 5:** Data and refinement statistics for glucose isomerase Raw data have been deposited in the Integrated Resource for Reproducibility in Macromolecular Crystallography (Grabowski *et al.*, 2016 ▸; https://proteindiffraction.org) at https://dx.doi.org/10.18430/m3.irrmc.5324.

Domain	1	2	3	1 + 2	1 + 2 + 3	Detwinned
PDB code	6oqz	6oqz	6oqz	6oqz	6oqz	6oqz
Space group	*I*222	*I*222	*I*222	*I*222	*I*222	*I*222
*a* (Å)	92.93 (9)	92.93 (9)	92.93 (9)	92.93 (9)	92.93 (9)	92.93 (9)
*b* (Å)	97.94 (10)	97.94 (10)	97.94 (10)	97.94 (10)	97.94 (10)	97.94 (10)
*c* (Å)	102.71 (10)	102.71 (10)	102.71 (10)	102.71 (10)	102.71 (10)	102.71 (10)
Mosaicity (°)	0.46	0.46	0.46	0.46	0.46	0.46
Resolution (Å)	1.6	1.6	1.6	1.6	1.6	1.6
*TWINABS* data statistics						
No. of data	237474	237699	237893	126763	10261	—
No. of unique data	47082	51266	48298	75981	7435	—
*I*/σ(*I*)	9.0	8.2	5.7	10.4	9.9	—
*R*_int_	0.0546	0.0572	0.0804	—	0.0592	—
*R*_r.i.m_ †	0.0594	0.0631	0.0885	—	0.0615	—
Fractional contribution	0.436	0.406	0.158	—	—	—
Overall *B* factor from Wilson plot (Å^2^)	8.62	8.6	7.0	9.38	9.52	9.03
*SHELXL*						
*R*1 [*I* > 4σ(*I*)]	0.133	0.131	0.129	0.146	0.149	0.177
Data used	73778	75962	73480	181519	229809	61943
Unique data used	51919	57462	52891	61334	61848	61943
Completeness (%)	83.7	92.7	85.3	98.9	99.8	99.9
No. of parameters	13377	13377	13377	13377	13377	13375
*wR*2 (all data)	0.360	0.358	0.357	0.402	0.411	0.452
*R*1 (after dispersion correction and merging)	0.155	0.158	0.157	0.177	0.181	0.191
*k*_2_	0.4259 (16)	0.393 (3)	0.416 (3)	0.4146 (13)	0.4183 (13)	—
*k*_3_	0.1622 (13)	0.167 (2)	0.1650 (15)	0.1696 (15)	0.1636 (8)	—
*R*1(free) (all 3101 data)						0.221
*R*1 (after dispersion correction and merging) against the detwinned data	0.199	0.198	0.202	0.189	0.188	—
Solvent content (%)	55	55	55	55	55	55
No. of non-H atoms						
Protein	3050	3050	3050	3050	3050	3050
Ion	2	2	2	2	2	2
MPD	8	8	8	8	8	8
Water	284	284	284	284	284	284
R.m.s.d., bonds (Å)	0.0102	0.0100	0.0097	0.0184	0.0213	0.0090
R.m.s.d., angles (°)	2.00	2.00	2.02	2.81	3.13	1.82
Average *B* factors (Å^2^)						
Main chain	13.56	13.38	13.34	13.73	13.54	13.53
Side chain and water	19.59	19.49	19.51	20.09	20.12	19.17
Ions	10.53	10.30	10.70	10.13	10.17	10.52
MPD	29.06	28.18	29.17	29.08	29.67	25.72
Ramachandran plot						
Most favoured (%)	96.85	96.59	97.11	97.11	96.85	97.11
Allowed (%)	2.62	2.89	2.36	2.36	2.62	2.36
Outliers (%)	0.52	0.52	0.52	0.52	0.52	0.52

† Calculated as [*N*/(*N* − 1)]^1/2^ × *R*_int_, where *N* is the data multiplicity.
