# Supplementary material for: Non-merohedral twinning: from minerals to proteins
Source: Acta Crystallogr D Struct Biol. 2019 Nov 19;75(Pt 12):1040–50. doi: 10.1107/S2059798319010179 (PMC6889912; doi:10.1107/S2059798319010179)
Supplement: Supplementary file 2 [file d-75-01040-sup2.pdf]

STRUCTURAL
BIOLOGY

Volume 75 (2019)

Supporting information for article:

Non-merohedral twinning: from minerals to proteins

Madhumati Sevvana, Michael Ruf, Isabel Usón, George Sheldrick and Regine Herbst-Irmer

S1. Main Principles

S1.1. HKLF5 Format

An overlapped reflection is described by its two (or more) components. Therefore there are two (or more) lines in the HKLF5 data file. The indices *h'k'l'* are the indices derived with orientation matrix 2, while *hkl* is derived with orientation matrix 1. The absolute value of the integer number in the last column, batch number, defines the domain. A negative number means that it overlaps with all following reflections until the batch number is positive. F^2 represents the summed intensity.

h'	k'	l'	F^2	$\sigma(F^2)$	-2
h	k	l	F^2	$\sigma(F^2)$	1

S1.2. Storage of Reflections

h	*k*	*l*		component	
1	-2	3	…	1	equivalent singles
-1	-2	-3	…	1	
-1	-2	-3	…	2	not equivalent to the above singles
-1	-2	-3	…	-2	equivalent groups
2	0	4	…	1	
1	2	-3	…	-2	
-2	0	-4	…	1	
5	1	1	…	-2	not equivalent to the other groups shown here
1	-2	-3	…	-3	
-1	1	2	…	1	

S2. Small Molecule Examples

S2.1. Chromite

S2.1.1. Determination of the Cell Constants and the Twin Law

S2.1.1.1 Output using default options in *CELL_NOW*

```
The following cells would appear to be plausible, but should be checked using
XPREP because they are not necessarily the conventional cells.

FOM, % within 0.2, a..gamma, volume and lattice type for potential unit-cells:

  1 1.000  85.3    5.874   5.879  14.405   90.06   89.97  120.35     429.3  P
  2 0.602  80.1    5.879   5.896  14.405   90.05   89.94  120.31     431.1  P
  3 0.590  83.6    5.874   5.896  14.405   90.05   90.03  119.33     435.0  P
  4 0.397  86.0    5.874  14.405   5.880   89.96  120.95   90.03     426.7  P
  5 0.243  57.2    8.308   8.312   8.323   90.02   90.11   89.98     574.8  F
  6 0.202  78.8    5.879  14.405   5.880   89.96  118.70   89.94     436.8  P

------------------------------------------------------------------------------

Cell for domain  1:    5.874    5.879   14.405    90.06    89.97   120.35

Figure of merit: 0.611   %(0.1):  59.7   %(0.2):  85.3   %(0.3):  97.0

Orientation matrix:  0.10209832  0.10334371 -0.05548610
                    -0.07077033 -0.16690505 -0.03656234
                    -0.15323937  0.01771556 -0.02008961

Percentages of reflections in this domain not consistent with lattice types:
A: 43.3,  B: 44.7,  C: 43.3,  I: 45.4,  F: 65.7,  O: 11.5  and  R: 49.5%

Percentages of reflections in this domain that do not have:
h=2n: 45.9,  k=2n: 44.5,  l=2n: 55.5,  h=3n: 65.7,  k=3n: 64.5,  l=3n: 61.1%

  434 reflections within tolerance assigned to domain  1,
  434 of them exclusively;   293 reflections not yet assigned to a domain
```

It is not obvious from this table that cell 5 is a good choice, but rotating the others did not index enough of the remaining reflections. To force *CELL_NOW* to find only the known F-centred cubic cell, the cell search was restricted. Then it just finds the two twin components:

S2.1.1.2 *CELL_NOW* Output Searching for vectors with 8.00 < d < 9.00

```
The following cells would appear to be plausible, but should be checked using
XPREP because they are not necessarily the conventional cells.

FOM, % within 0.2, a..gamma, volume and lattice type for potential unit-cells:

  1 1.000  57.2    8.308   8.312   8.323   89.98   90.11   90.02     574.8  F
  2 0.479  52.4    8.305   8.317   8.333   89.97   90.07   89.87     575.6  F

------------------------------------------------------------------------------

Cell for domain  1:    8.308    8.312    8.323    89.98    90.11    90.02

Figure of merit: 0.572   %(0.1):  53.9   %(0.2):  58.6   %(0.3):  67.5

Orientation matrix:  0.10682273 -0.05521402  0.00478703
                     0.00018990  0.01029280  0.11971127
                    -0.05545969 -0.10638566  0.00915069
```

```
Percentages of reflections in this domain not consistent with lattice types:
A:  0.5,  B:  0.5,  C:  0.0,  I: 55.9,  F:  0.5,  O: 63.8  and  R: 60.5%

Percentages of reflections in this domain that do not have:
h=2n: 56.4,  k=2n: 56.4,  l=2n: 55.9,  h=3n: 66.6,  k=3n: 67.3,  l=3n: 64.3%

  392 reflections within tolerance assigned to domain  1,
  392 of them exclusively;   335 reflections not yet assigned to a domain

-------------------------------------------------------------------------------

Cell for domain  2:    8.308    8.312    8.323    89.98    90.11    90.02

Figure of merit: 0.959   %(0.1):  94.0   %(0.2):  94.6   %(0.3):  95.5

Orientation matrix:  0.05205004  0.10830130 -0.00625146
                     0.08783259 -0.04612121 -0.06782782
                    -0.06374275  0.02482138 -0.09898356

Rotated from first domain by 144.5 degrees about
reciprocal axis  1.000  0.359  0.359  and real axis  1.000  0.359  0.359

Twin law to convert hkl from first to      0.628   0.331   0.704
this domain (SHELXL TWIN matrix):          0.704  -0.628  -0.330
                                           0.332   0.704  -0.629
```

S2.1.1.3 Symmetry allowed Rotation of the Second Domain

In the cubic crystal system, there are many symmetry equivalent twin laws. To show that this rotation of 144.5 ° is a proper rotation we applied a rotation of -90 ° around 0 1 0, which leads to a symmetry equivalent cell in Laue group $m\bar{3}m$. This operation was applied with the program *GEMINI* (Sparks, 2000).

```
  A1 [Orientation matrix] =
    0.05206   0.10830  -0.00625
    0.08781  -0.04612  -0.06785
   -0.06376   0.02484  -0.09897
  unit cell parameters =
    8.3085    8.3123    8.3224    89.976    90.107    90.018
  A2 [Orientation matrix] =
   -0.00625   0.10830  -0.05206
   -0.06785  -0.04612  -0.08781
   -0.09897   0.02484   0.06376
  unit cell parameters =
    8.3224    8.3123    8.3085    89.982    89.893    89.976
  A2(inverse) * A1 [transforms h1 to h2] =
    0.00000   0.00000   1.00000
    0.00000   1.00000   0.00000
   -1.00000   0.00000   0.00000
<<< angle of rotation around   0.00  1.00  0.00 >>> =
  -89.99998
```

The rotation between this cell and the first cell is a rotation of 180 ° around -2 -1 1

```
  A1 [Orientation matrix] =
    0.10682  -0.05521   0.00479
    0.00019   0.01029   0.11971
   -0.05546  -0.10639   0.00915
  unit cell parameters =
    8.3083    8.3125    8.3225    89.978    90.105    90.016
  A2 [Orientation matrix] =
   -0.00625   0.10830  -0.05206
   -0.06785  -0.04612  -0.08781
   -0.09897   0.02484   0.06376
  unit cell parameters =
    8.3225    8.3125    8.3083    89.984    89.895    89.978
  A2(inverse) * A1 [transforms h1 to h2] =
    0.33218   0.70391  -0.62884
    0.70352  -0.62839  -0.33038
   -0.62830  -0.33110  -0.70382
<<< angle of rotation around  -2.00 -1.06  0.94 >>> =
 -179.98076
```

S2.1.2. Data Scaling, Absorption Correction and Merging in *TWINABS*

```
      675 data (     69 unique ) involve domain  1 only,  mean I/sigma  60.4
      659 data (     69 unique ) involve domain  2 only,  mean I/sigma  51.9
       45 data (     19 unique ) involve   2 domains,  mean I/sigma  82.4

Unique HKLF 4 data extracted from all observed data

 Cycle   N(1)  Rint(1)   N(all)  Rint(all)  Twin fractions
    1     706   0.0360    1343   0.0546   0.5749   0.4251
    2     706   0.0446    1343   0.0491   0.5740   0.4260
    3     706   0.0442    1343   0.0490   0.5740   0.4260
    4     706   0.0440    1343   0.0490   0.5740   0.4260
    5     706   0.0440    1343   0.0490   0.5740   0.4260
    6     706   0.0439    1343   0.0489   0.5740   0.4260
    7     706   0.0439    1343   0.0489   0.5740   0.4260
    8     706   0.0439    1343   0.0489   0.5740   0.4260
    9     706   0.0439    1343   0.0489   0.5740   0.4260
   10     706   0.0439    1343   0.0489   0.5740   0.4260
   11     706   0.0439    1343   0.0489   0.5740   0.4260
   12     706   0.0439    1343   0.0489   0.5740   0.4260
   13     706   0.0439    1343   0.0489   0.5740   0.4260
   14     706   0.0439    1343   0.0489   0.5740   0.4260
   15     706   0.0439    1343   0.0489   0.5740   0.4260
   16     706   0.0439    1343   0.0489   0.5740   0.4260
   17     706   0.0439    1343   0.0489   0.5740   0.4260
   18     706   0.0439    1343   0.0489   0.5740   0.4260
   19     706   0.0439    1343   0.0489   0.5740   0.4260
   20     706   0.0439    1343   0.0489   0.5740   0.4260

 N(1) and Rint(1) refer to singles and composites that include domain 1.

 Rint = 0.0489  for all    1343  observations and
 Rint = 0.0484  for all    1107  observations with I > 3sigma(I)
```

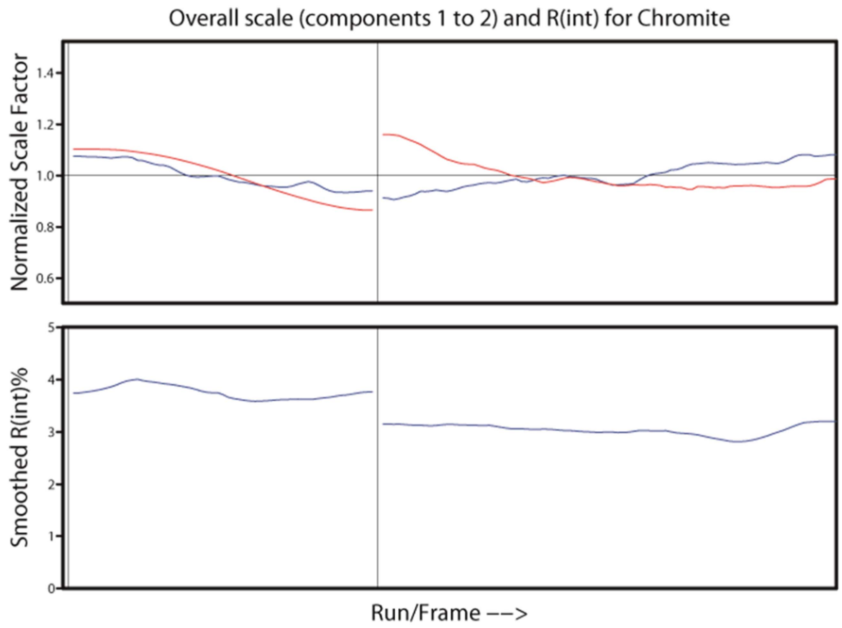

Figure S1 Normalized scale factor and smoothed R_{int} per frame, blue component 1 and red component 2.

S2.1.3. Space Group Determination using the Program *XPREP*

```
--------------------------------------------------------------------------------

SPACE GROUP DETERMINATION

Lattice exceptions:  P      A      B      C      I      F     Obv    Rev    All

N (total) =          0      0      0      0     30      0     48     45     69
N (int>3sigma) =     0      0      0      0     28      0     42     41     63
Mean intensity =   0.0    0.0    0.0    0.0   64.2    0.0   97.0   73.3   81.7
Mean int/sigma =   0.0    0.0    0.0    0.0   78.1    0.0   66.1   67.4   65.7


Crystal system C and Lattice type F selected

Mean |E*E-1| = 0.674 [expected .968 centrosym and .736 non-centrosym]


Systematic absence exceptions:

        41/43  d--   --c

N          3    11    26
N I>3s     2     7    25
<I>      1.1   0.7  84.5
<I/s>   15.7  11.1  71.0


Identical indices and Friedel opposites combined before calculating R(sym)

Option  Space Group  No.  Type  Axes  CSD  R(sym) N(eq)  Syst. Abs.   CFOM

[A] Fm-3          #202  centro   1     1  0.000      0  0.0 / 11.1  59.56
[B] F23           #196  chiral   1     1  0.000      0  0.0 / 11.1  51.29
[C] F-43m         #216  non-cen  1     3  0.000      0  0.0 / 11.1  26.29
[D] Fm-3m         #225  centro   1    27  0.000      0  0.0 / 11.1  13.13
[E] F432          #209  chiral   1     2  0.000      0  0.0 / 11.1  34.62
[F] F4(1)32       #210  chiral   1     5  0.000      0  0.0 / 11.1  17.95
```

XPREP does not find the right space group because the reflections that should be absent for the d-glide plane are not weak enough, but *SHELXT* that uses phases rather than systematic absences convincingly finds the correct space group $Fd\bar{3}m$.

S2.1.4. Structure Solution with *SHELXT*

Try	N(iter)	CC	R(weak)	CHEM	CFOM	best	Sig(min)	N(F1)	Vol/N
1	100	95.09	0.0231	0.0000	0.9278	0.9278	3.711	11	13.02
2	100	95.07	0.0267	0.0000	0.9240	0.9278	3.541	11	13.02
3	100	91.74	0.0647	0.0000	0.8527	0.9278	3.534	11	13.02
4	100	95.08	0.0257	0.0000	0.9251	0.9278	3.945	11	13.02
5	100	94.84	0.0172	0.0000	0.9312	0.9312	6.683	10	14.33
6	100	94.01	0.0629	0.0000	0.8771	0.9312	6.497	10	14.33
7	100	95.07	0.0228	0.0000	0.9279	0.9312	3.716	11	13.02
8	100	94.47	0.0622	0.0000	0.8825	0.9312	2.968	11	13.02

8 attempts, solution 5 selected with best CFOM = 0.9312, Alpha0 = 0.115

Structure solution: 0.089 secs

4 Centrosymmetric and 4 non-centrosymmetric space groups evaluated

Space group determination: 0.320 secs

R1	Rweak	Alpha	SysAbs	Orientation	Space group	Flack_x	File	Formula
**0.046**	**0.001**	**0.059**	**10.28**	**as input**	**Fd-3m**		**twin4_a**	**O4 Cr Fe2**
0.052	0.001	0.060	15.72	as input	F4(1)32	no Fp	twin4_b	O4 Cr Fe2
0.072	0.001	0.059	0.00	as input	F-43m	no Fp	twin4_c	O4 Cr Fe2

S2.1.5. Structure Refinement

Table S1 Data for Chromite

Identification code	detwinned	domain1	domain2	both domains
Empirical formula		$Cr_2\ Fe_{0.66}\ Mg_{0.34}\ O_4$		
Formula weight		213.22		
Temperature		292(2) K		
Wavelength		0.71073 Å		
Crystal system		Cubic		
Space group		$Fd\bar{3}m$		
Unit cell dimensions		a = 8.321(2) Å		
Volume [Å^3]		576.1(4)		
Z		8		
Density (calculated) [Mg/m^3]		4.916		
Absorption coefficient [mm^{-1}]		10.635		
F(000)		810		
Theta range for data collection		4.242 to 30.352°.		
Index ranges		-11<=h<=9, -11<=k<=11, -8<=l<=11		
Reflections collected	1343	706	682	1343
Independent reflections	60	60	60	60
R_{int}	0.0489	0.0439	0.0489	0.0489
Completeness to θ = 25.242°	97.4 %	97.4 %	97.4 %	97.4 %
Refinement method		Full-matrix least-squares on F^2		
Data / restraints / parameters	60 / 0 / 9	60 / 0 / 10	60 / 0 / 10	138 / 0 / 10
Goodness-of-fit on F^2	1.408	1.275	1.287	1.169
$R1$ [I > 2 σ(I)]	0.0161	0.0189	0.0271	0.0264
$wR2$ [I > 2 σ(I)]	0.0434	0.0510	0.0694	0.0672
$R1$ (all data)	0.0171	0.0200	0.0276	0.0273
$wR2$ (all data)	0.0441	0.0521	0.0697	0.0680
Extinction coefficient	0.0042(8)	0.0096(17)	0.006(2)	0.008(2)
Largest diff. peak [e·Å^{-3}]	0.444	0.519	0.694	0.561
Largest diff. hole	-0.416	-0.588	-0.807	-0.505

S2.2. $Cp^*_2MeZrOTiMe_2Cp^*$

S2.2.1. Determination of the Cell Constants and the Twin Law

```
The following cells would appear to be plausible, but should be checked using
XPREP because they are not necessarily the conventional cells.

FOM, % within 0.2, a..gamma, volume and lattice type for potential unit-cells:

  1 1.000  54.6    8.676  15.514  11.578   89.93   94.47   89.87    1553.7  I
  2 0.846  54.2   13.923  15.514   8.676   89.87  123.97   90.18    1554.2  C
  3 0.723  60.0   23.245  30.889   8.676   90.06   94.46   90.05    6210.8  C?
  4 0.720  60.0    8.676  15.514  23.168   90.02   94.49   90.13    3108.8  P
  5 0.678  59.6   23.168  31.009   8.676   90.11   94.49   90.05    6213.8  C?
  6 0.583  58.4    8.676  15.452  23.245   90.03   94.46   90.07    3106.8  P
  7 0.540  55.6    8.676  10.399  10.434   96.13  111.98  111.85     776.5  P
  8 0.535  56.0    8.676  10.399  10.782   66.27   63.70   68.15     775.8  P
  9 0.527  60.4    8.676  30.889  24.180   89.93  106.50   90.06    6213.5  I?
 10 0.502  55.6    8.676  10.434  10.784   66.16   63.54   68.02     776.6  P
```

…

```
168 0.203  57.6    8.676  10.399  34.022  113.77  103.29  111.85    2332.0  P
169 0.202  58.2    8.676  10.399  35.474   90.14  125.05  111.85    2331.6  P
170 0.201  55.6    8.676  10.434  29.524   70.55   78.80   68.02    2329.7  P
171 0.200  54.6   11.578  15.514  43.371   90.12   94.48   90.07    7766.9  I
172 0.200  64.3    8.676  21.564  41.596   66.30   68.14   63.70    6206.4  P?

------------------------------------------------------------------------------

Cell for domain  1:    8.676   15.514   11.578    89.93    94.47    89.87

Figure of merit: 0.596   %(0.1):  51.6   %(0.2):  58.2   %(0.3):  68.3

Orientation matrix:  0.01542265 -0.00931260  0.08560041
                    -0.10873301  0.01906555  0.00839843
                    -0.03612697 -0.06086576 -0.01034160

Percentages of reflections in this domain not consistent with lattice types:
A: 52.4,  B: 51.4,  C: 51.4,  I:  6.2,  F: 77.6,  O: 64.1  and  R: 63.8%

Percentages of reflections in this domain that do not have:
h=2n: 50.3,  k=2n: 52.8,  l=2n: 48.6,  h=3n: 68.6,  k=3n: 64.1,  l=3n: 69.3%

  290 reflections within tolerance assigned to domain  1,
  290 of them exclusively;   208 reflections not yet assigned to a domain

------------------------------------------------------------------------------

Cell for domain  2:    8.676   15.514   11.578    89.93    94.47    89.87

Figure of merit: 0.790   %(0.1):  69.2   %(0.2):  69.2   %(0.3):  71.6

Orientation matrix: -0.00556339  0.06369888  0.01218753
                     0.11233460  0.00065195  0.02694662
                     0.02675576  0.00984316 -0.08142637

Rotated from first domain by 180.0 degrees about
reciprocal axis  0.000  1.000  0.995  and real axis  0.101  0.556  1.000

Twin law to convert hkl from first to     -1.000   0.000  -0.001
this domain (SHELXL TWIN matrix):          0.130  -0.283   1.290
                                           0.131   0.713   0.283

  285 reflections within tolerance assigned to domain  2,
  144 of them exclusively;    64 reflections not yet assigned to a domain

------------------------------------------------------------------------------

Cell for domain  3:    8.676   15.514   11.578    89.93    94.47    89.87

Figure of merit: 0.286   %(0.1):  25.0   %(0.2):  42.2   %(0.3):  65.6
```

```
Orientation matrix:  0.00777858 -0.06386182  0.01084856
                    -0.10746484 -0.00107078  0.02552981
                    -0.04191285 -0.00868228 -0.08206978

Rotated from first domain by  74.6 degrees about
reciprocal axis  1.000 -0.015 -0.091  and real axis  1.000 -0.006  0.007

Twin law to convert hkl from first to      1.000  -0.009  -0.002
this domain (SHELXL TWIN matrix):         -0.129   0.267  -1.296
                                          -0.057   0.718   0.264

  142 reflections within tolerance assigned to domain  3,
   27 of them exclusively;    37 reflections not yet assigned to a domain

-----------------------------------------------------------------------------

Cell for domain  4:    8.676   15.514   11.578    89.93    94.47    89.87

Figure of merit: 0.256   %(0.1):  13.5   %(0.2):  29.7   %(0.3):  43.2

Orientation matrix: -0.00058412  0.00925286  0.08542314
                     0.11036003 -0.01910612  0.01058073
                     0.03444131  0.06086215 -0.00979007

Rotated from first domain by 180.0 degrees about
reciprocal axis -0.010  0.001  1.000  and real axis  0.087  0.000  1.000

Twin law to convert hkl from first to     -1.002   0.000  -0.020
this domain (SHELXL TWIN matrix):          0.001  -1.000   0.002
                                           0.174  -0.001   1.002

  195 reflections within tolerance assigned to domain  4,
   15 of them exclusively;    22 reflections not yet assigned to a domain
```

Cell number 4 chosen from list

```
-----------------------------------------------------------------------------

Cell for domain  1:    8.676   15.514   23.168    90.02    94.49    90.13

Figure of merit: 0.596   %(0.1):  56.4   %(0.2):  60.0   %(0.3):  64.5

Orientation matrix: -0.01547039 -0.00926021 -0.04278166
                     0.10872833  0.01907069 -0.00419740
                     0.03613273 -0.06087209  0.00516856

Percentages of reflections in this domain not consistent with lattice types:
A: 50.8,  B: 48.8,  C: 52.2,  I: 50.8,  F: 75.9,  O: 68.2  and  R: 66.9%

Percentages of reflections in this domain that do not have:
h=2n: 49.5,  k=2n: 54.2,  l=2n: 13.4,  h=3n: 68.9,  k=3n: 66.2,  l=3n: 70.9%

  299 reflections within tolerance assigned to domain  1,
  299 of them exclusively;   199 reflections not yet assigned to a domain

-----------------------------------------------------------------------------

Cell for domain  2:    8.676   15.514   23.168    90.02    94.49    90.13

Figure of merit: 0.950   %(0.1): 100.0   %(0.2): 100.0   %(0.3): 100.0

Orientation matrix:  0.00558704  0.06371026 -0.00606874
                    -0.11235817  0.00069606 -0.01345354
                    -0.02666801  0.00976586  0.04070350

Rotated from first domain by 179.9 degrees about
reciprocal axis  0.000 -0.503  1.000  and real axis -0.183  1.000 -0.897

Twin law to convert hkl from first to     -1.000   0.000   0.000
this domain (SHELXL TWIN matrix):         -0.130  -0.281  -0.645
                                           0.264  -1.428   0.281

  306 reflections within tolerance assigned to domain  2,
  199 of them exclusively;     0 reflections not yet assigned to a domain

----------------------------------------------------------------------
```

S2.3. Data Scaling, Absorption Correction and Merging in *TWINABS*

```
    30843 data (   5738 unique ) involve domain  1 only,  mean I/sigma   3.1
    30852 data (   5739 unique ) involve domain  2 only,  mean I/sigma   2.9
     4354 data (   1814 unique ) involve   2 domains,  mean I/sigma   4.8

Unique HKLF 4 data extracted from all observed data

 Cycle   N(1) Rint(1)  N(all) Rint(all) Twin fractions
    1   35149  0.0974   65953  0.1069   0.5328  0.4672
    2   35149  0.0951   65953  0.0977   0.5321  0.4679
    3   35149  0.0951   65953  0.0976   0.5320  0.4680
    4   35149  0.0951   65953  0.0976   0.5320  0.4680
    5   35149  0.0951   65953  0.0976   0.5320  0.4680
    6   35149  0.0951   65953  0.0976   0.5320  0.4680
    7   35149  0.0951   65953  0.0976   0.5320  0.4680
    8   35149  0.0951   65953  0.0976   0.5320  0.4680
    9   35149  0.0951   65953  0.0976   0.5320  0.4680
   10   35149  0.0951   65953  0.0976   0.5320  0.4680
   11   35149  0.0951   65953  0.0976   0.5320  0.4680
   12   35149  0.0951   65953  0.0976   0.5320  0.4680
   13   35149  0.0951   65953  0.0976   0.5320  0.4680
   14   35149  0.0951   65953  0.0976   0.5320  0.4680
   15   35149  0.0951   65953  0.0976   0.5320  0.4680
   16   35149  0.0951   65953  0.0976   0.5320  0.4680
   17   35149  0.0951   65953  0.0976   0.5320  0.4680
   18   35149  0.0951   65953  0.0976   0.5320  0.4680
   19   35149  0.0951   65953  0.0976   0.5320  0.4680
   20   35149  0.0951   65953  0.0976   0.5320  0.4680

 N(1) and Rint(1) refer to singles and composites that include domain 1.

 Rint = 0.0976  for all   65953  observations and
 Rint = 0.0691  for all   21961  observations with I > 3sigma(I)

 Rint is based on agreement between observed single and composite intensities
 and those calculated from refined unique intensities and twin fractions.
```

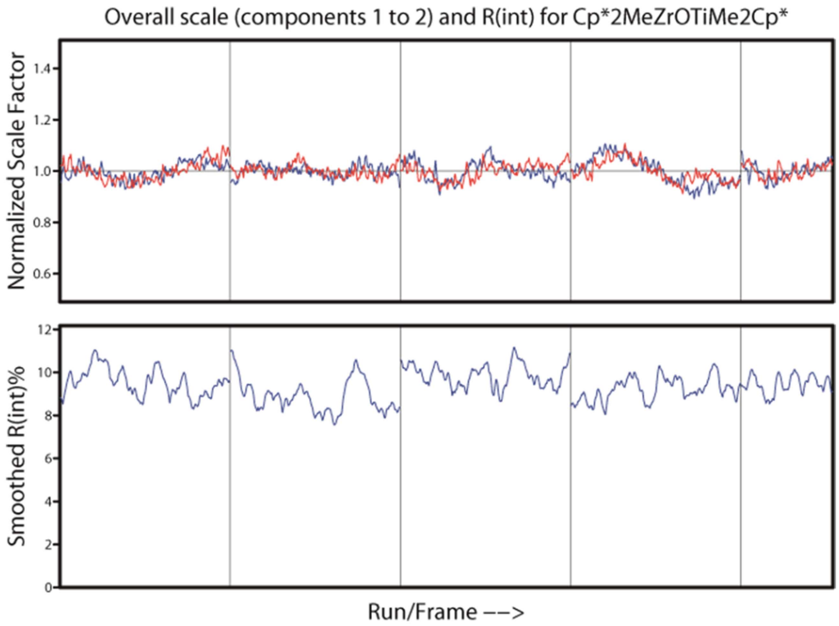

Figure S2 Normalized scale factor and smoothed R_{int} per frame, blue component 1 and red component 2.

S2.4. Space Group Determination using the Program *XPREP*

```
SPACE GROUP DETERMINATION

Lattice exceptions:  P      A      B      C      I      F     Obv    Rev    All

N (total) =          0   2896   2898   2904   2903   4349   3875   3872   5805
N (int>3sigma) =     0   2272   2347   2329   2337   3474   3124   3142   4685
Mean intensity =   0.0   11.2   12.3   11.8   11.9   11.8   12.1   11.6   12.0
Mean int/sigma =   0.0   13.6   13.8   13.8   13.8   13.7   13.7   13.7   13.8

Crystal system M and Lattice type P selected

Mean |E*E-1| = 0.961 [expected .968 centrosym and .736 non-centrosym]

Chiral flag NOT set


Systematic absence exceptions:

        -21-   -a-   -c-   -n-

N         9   215   207   214
N I>3s    2    97     2    99
<I>     0.5  22.4   0.2  22.5
<I/s>   2.7  10.6   1.0  10.7


Identical indices and Friedel opposites combined before calculating R(sym)

Option  Space Group  No.  Type  Axes  CSD  R(sym) N(eq)  Syst. Abs.   CFOM

[A] P2(1)/c        # 14  centro   1 19410  0.000      0  2.7 / 10.6   1.14
```

Here *XPREP* convincingly finds *P*2_1/*c* but as *SHELXT* shows, the correct space group is *Pc*. The *Flack* parameter cannot be used as a check because the data had been merged in the wrong space group.

S2.5. Structure Solution with *SHELXT*

```
 Try N(iter)  CC   R(weak)   CHEM    CFOM    best  Sig(min) N(P1) Vol/N
   1   100   92.96  0.0491  0.5784  0.8805  0.8805  2.390   145   20.98
   2   100   93.62  0.0586  0.8534  0.8776  0.8805  2.453   158   19.25
   3   100   93.33  0.0551  0.8889  0.8782  0.8805  2.469   151   20.15
   4   100   90.46  0.0483  0.5725  0.8562  0.8805  2.460   164   18.55
   5   100   92.61  0.0595  0.6771  0.8665  0.8805  2.472   141   21.58
   6   100   92.94  0.0701  0.6887  0.8593  0.8805  2.470   165   18.44
   7   100   93.83  0.0501  0.6577  0.8882  0.8882  2.424   169   18.00
   8   100   92.27  0.0817  0.8557  0.8409  0.8882  2.235   149   20.42

      8 attempts, solution  7 selected with best CFOM = 0.8882, Alpha0 = 0.193

 Structure solution:       0.802 secs

   8 Centrosymmetric and   6 non-centrosymmetric space groups evaluated

 Space group determination:       0.117 secs

   R1  Rweak Alpha SysAbs  Orientation  Space group  Flack_x  File    Formula
 0.072 0.010 0.023 1.01     as input    Pc           no Fp   twin4_a  C66 O2 Ti2 Zr2
 0.286 0.033 0.132 1.08     as input    P2(1)/c              twin4_b  C59 O8 Ti2 Zr
```

S2.6. Structure Refinement with *SHELXL*

Table S2 Data for $Cp^*_2MeZrOTiMe_2Cp^*$

Domain	detwinned	domain 1	domain 2	both domains
Emp. formula	C_{33} H_{54} O Ti Zr			
Formula weight	605.88			
Temperature	100(2) K			
Wavelength	0.71073 Å			
Cryst. Syst.	Monoclinic			
Space group	*Pc*			
Unit cell dimensions	*a* = 8.627(2) Å			
	b = 15.358(2) Å			
	c = 23.033(3) Å			
	β = 94.45(2)°.			
Volume	3042.5(9) $Å^3$			
Z	4			
Dens (calc.)	1.323 Mg/m^3			
Absorption coeff. [mm^{-1}]	0.628			
F(000)	1288			
Theta range	1.326 to 25.362°			
Index ranges	-10<=h<=10, -18<=k<=18, -27<=l<=27			
Refl. coll.	65953	35149	35030	65953
Ind. refl.	11172	11343	11325	24945
R_{int}	0.0976	0.0951	0.0992	0.0976
Compl. theta = 25.242°	100.0 %	100.0 %	100.0 %	100.0 %
data	11172	11343	11325	24945
restraints	980	980	980	980
parameters	685	686	686	686
GooF on F^2	1.056	1.071	1.070	1.081
*R*1 [I > 2 σ(I)]	0.0481	0.0581	0.0588	0.0624
*wR*2 [I > 2 σ(I)]	0.0959	0.1155	0.1137	0.1186
*R*1 (all data)	0.0588	0.0718	0.0738	0.0814
*wR*2 (all data)	0.1019	0.1260	0.1246	0.1314
Flack para.	0.47(2)	0.02(3)	0.01(3)	0.02(2)
Largest diff. peak [e·$Å^{-3}$]	0.595	0.822	0.856	1.006
Largest diff. hole [e·$Å^{-3}$]	-0.386	-0.460	-0.481	-0.574

S2.7. Pseudo-Symmetry

Table S3 Atomic coordinates of the metal atoms

Atom	x	y	z
Zr1	-0.352	0.505	0.056
Ti1	-0.140	0.516	0.204
Zr2	0.138	0.012	0.296
Ti2	0.352	0.011	0.444

The coordinates of Zr1/Ti2 and Zr2/Ti1 are related by the symmetry operator –x, y+0.5, 0.5-z describing a 2_1 axis. The coordinates of the two Zr atoms as well the two Ti atoms are related by x+0.5, y+0.5, z+0.25 describing an I-centring for a cell with a halved *c* axis.

S3. Protein Examples

S3.1. Cubic Insulin

S3.1.1. Cell Determination with *CELL_NOW*

```
The following cells would appear to be plausible, but should be checked using
 XPREP because they are not necessarily the conventional cells.

 FOM, % within 0.2, a..gamma, volume and lattice type for potential unit-cells:

   1 1.000  58.5   78.784  79.497  79.551   89.09   90.92   90.47  498090.2  I
   2 0.812  44.2   78.664  78.917  80.213   89.82   90.73   90.29  497908.0  I

 ------------------------------------------------------------------------------

 Cell for domain  1:   78.784   79.497   79.551    89.09    90.92    90.47

 Figure of merit: 0.529   %(0.1):  56.6   %(0.2):  60.0   %(0.3):  66.0

 Orientation matrix: -0.00341436 -0.00699369 -0.00984969
                      0.00029053  0.01024655 -0.00744876
                      0.01222377 -0.00209318 -0.00236595

 Percentages of reflections in this domain not consistent with lattice types:
 A: 52.0,  B: 48.9,  C: 50.2,  I:  4.0,  F: 75.6,  O: 67.6  and  R: 68.5%

 Percentages of reflections in this domain that do not have:
 h=2n: 51.8,  k=2n: 48.6,  l=2n: 50.2,  h=3n: 67.3,  k=3n: 66.4,  l=3n: 66.8%

  1495 reflections within tolerance assigned to domain  1,
  1495 of them exclusively;   912 reflections not yet assigned to a domain

 ------------------------------------------------------------------------------

 Cell for domain  2:   78.784   79.497   79.551    89.09    90.92    90.47

 Figure of merit: 0.667   %(0.1):  71.9   %(0.2):  99.6   %(0.3):  99.7

 Orientation matrix:  0.00032865  0.01016351  0.00724939
                     -0.01269073  0.00017090 -0.00002355
                     -0.00001322 -0.00741341  0.01027346

 Rotated from first domain by 177.0 degrees about
 reciprocal axis  1.000 -0.833  0.617  and real axis  1.000 -0.818  0.632

 Twin law to convert hkl from first to     -0.035  -0.811   0.581
 this domain (SHELXL TWIN matrix):         -0.781  -0.341  -0.544
                                            0.626  -0.451  -0.622
```

S3.1.2. Excerpt of the *TWINABS* Output for Insulin

```
   202583 data (  11532 unique ) involve domain  1 only,  mean I/sigma   9.4
   202218 data (  11502 unique ) involve domain  2 only,  mean I/sigma   7.5
    29318 data (  19152 unique ) involve   2 domains,  mean I/sigma  10.2

Unique HKLF 4 data extracted from all observed data

 Cycle   N(1) Rint(1)  N(all) Rint(all) Twin fractions
    1  231588  0.0719  433427  0.3847   0.8909  0.1091
    2  231627  0.1513  433710  0.3956   0.8093  0.1907
    3  231627  0.1949  433710  0.4025   0.7707  0.2293
    4  231627  0.2355  433710  0.4100   0.7375  0.2625
    5  231627  0.2704  433710  0.4164   0.7099  0.2901
    6  231627  0.2991  433710  0.4214   0.6875  0.3125
```

```
   7  231627  0.3222  433710  0.4251   0.6695  0.3305
   8  231627  0.3407  433710  0.4279   0.6551  0.3449
   9  231627  0.3550  433710  0.4298   0.6438  0.3562
  10  231627  0.3661  433710  0.4311   0.6350  0.3650
  11  231627  0.3746  433710  0.4321   0.6280  0.3720
  12  231627  0.3812  433710  0.4328   0.6225  0.3775
  13  231627  0.3863  433710  0.4333   0.6183  0.3817
  14  231627  0.3902  433710  0.4336   0.6149  0.3851
  15  231627  0.3933  433710  0.4339   0.6123  0.3877
  16  231627  0.3956  433710  0.4341   0.6103  0.3897
  17  231627  0.3975  433710  0.4343   0.6087  0.3913
  18  231627  0.3989  433710  0.4344   0.6075  0.3925
  19  231627  0.4000  433710  0.4345   0.6066  0.3934
  20  231627  0.4008  433710  0.4346   0.6058  0.3942

 N(1) and Rint(1) refer to singles and composites that include domain 1.

 Rint = 0.4346  for all  433710  observations and
 Rint = 0.4269  for all  291717  observations with I > 3sigma(I)

 Rint is based on agreement between observed single and composite intensities
 and those calculated from refined unique intensities and twin fractions.

 ** Warning: components may be inconsistently indexed, try reindex option.
```

Here it is necessary to re-index the second component because, as the R_{int} values indicate, the two components are indexed inconsistently. Note also that the systematic absences are the same for space group *I*23 and $I2_13$. *SHELXD* finds the six sulfur atoms using the anomalous differences, thereby confirming $I2_13$.

```
   11680 Corrected reflections written to file twin4.hkl
 Reflections merged according to point-group m-3
 Minimum and maximum apparent transmission:  0.646344  0.745992
 Additional spherical absorption correction applied with mu*r =  0.2000

 Indices of component  2 reindexed using matrix:   0  1  0  1  0  0  0  0 -1

 Unique HKLF 4 data extracted from all observed data

 Cycle   N(1) Rint(1)  N(all) Rint(all)  Twin fractions
    1  231588  0.0335  433429  0.0351   0.5809  0.4191
    2  231627  0.0336  433710  0.0347   0.5808  0.4192
    3  231627  0.0336  433710  0.0347   0.5808  0.4192
    4  231627  0.0336  433710  0.0347   0.5808  0.4192
    5  231627  0.0336  433710  0.0347   0.5808  0.4192
    6  231627  0.0336  433710  0.0347   0.5808  0.4192
    7  231627  0.0336  433710  0.0347   0.5808  0.4192
    8  231627  0.0336  433710  0.0347   0.5808  0.4192
    9  231627  0.0336  433710  0.0347   0.5808  0.4192
   10  231627  0.0336  433710  0.0347   0.5808  0.4192
   11  231627  0.0336  433710  0.0347   0.5808  0.4192
   12  231627  0.0336  433710  0.0347   0.5808  0.4192
   13  231627  0.0336  433710  0.0347   0.5808  0.4192
   14  231627  0.0336  433710  0.0347   0.5808  0.4192
   15  231627  0.0336  433710  0.0347   0.5808  0.4192
   16  231627  0.0336  433710  0.0347   0.5808  0.4192
   17  231627  0.0336  433710  0.0347   0.5808  0.4192
   18  231627  0.0336  433710  0.0347   0.5808  0.4192
   19  231627  0.0336  433710  0.0347   0.5808  0.4192
   20  231627  0.0336  433710  0.0347   0.5808  0.4192

 N(1) and Rint(1) refer to singles and composites that include domain 1.

 Rint = 0.0347  for all  433710  observations and
 Rint = 0.0290  for all  291717  observations with I > 3sigma(I)
```

S3.1.3. Excerpt of the *XPREP* Output for Insulin

```
SPACE GROUP DETERMINATION


Lattice exceptions:  P      A      B      C      I      F     Obv    Rev    All

N (total) =          0  10932  11206  11086      0  16612  14806  14817  22188
N (int>3sigma) =     0   8798   9087   8877      0  13381  11956  11884  17850
Mean intensity =   0.0    7.7    7.8    7.7    0.0    7.8    8.1    8.0    7.9
Mean int/sigma =   0.0   45.3   45.9   45.2    0.0   45.5   45.9   45.7   45.5


Crystal system C and Lattice type I selected

Mean |E*E-1| = 0.736 [expected .968 centrosym and .736 non-centrosym]

Chiral flag NOT set



Systematic absence exceptions:

        41/43  a--   --d

N         13   493   725
N I>3s    11   376   598
<I>     28.8  11.6  15.3
<I/s>   85.9  61.5  69.6


Identical indices and Friedel opposites combined before calculating R(sym)

Option  Space Group  No.  Type  Axes  CSD  R(sym) N(eq)  Syst. Abs.   CFOM

[A] Im-3           #204  centro   1     4  0.000      0  0.0 / 45.5  25.60
[B] I23            #197  chiral   1     4  0.000      0  0.0 / 45.5  20.22
[C] I2(1)3         #199  chiral   1     1  0.000      0  0.0 / 45.5  50.22

Estimation of SAD delta-F values

Local scaling employed with radius of 0.2119 reciprocal Angstroms


 Anomalous signal/noise ratios (1.0 is random).  The first line is based on

 input sigmas, the second on variances of F+ and F- (if not already averaged):

 Inf - 8.0 - 6.0 - 5.0 - 4.0 - 3.5 - 3.0 - 2.5 - 2.3 - 2.1 - 1.9 - 1.7 - 1.5 A

    8.43 10.52  8.75  7.01  3.88  3.30  2.19  1.80  1.51  1.27  1.08  0.99

    70.2  Neighbors used on average for F+/F- local scaling
 Ranom =   0.0361  before and   0.0363  after local scaling
```

S3.1.4. Substructure Solution in *SHELXD*

```
PSUM 17.00  PSMF Peaks: 165 120 45 32 23 20 20 18 17 17 15 6 6 5
 Cycle 18  Peaks 99 90 89 88 84 83 41 14
 R = 0.333, Min.fun. = 0.584, <cos> = 0.231, Ra = 0.426

    x        y        z       sof  height
 0.50729 0.16689 0.23044  1.000  99.90
 0.48027 0.07191 0.32732  1.000  91.00
 0.46030 0.09124 0.34034  1.000  90.16
 0.52850 0.16754 0.25194  1.000  88.85
 0.56024 0.13937 0.13426  1.000  85.24
 0.58659 0.14112 0.14703  1.000  83.94
 0.84604 0.34604 0.15396  0.333  41.54
 0.75000 0.36057 0.00000  0.500  14.07

 Minimum distances (top row, 0 if special position) and PSMF (bottom row)

 Peak    x      y      z      self  cross-vectors

 99.9   0.5073 0.1669 0.2304   13.0
                                9.5

 91.0   0.4803 0.0719 0.3273   16.5  10.8
                               16.9  19.0

 90.2   0.4603 0.0912 0.3403   19.4  11.0  2.4
                               19.1  13.9  4.7

 88.8   0.5285 0.1675 0.2519   13.6   2.4 10.2 10.5
                                5.9  13.3 16.1 20.4

 85.2   0.5602 0.1394 0.1343   19.6   8.8 13.7 11.5  9.8
                               11.7  14.4  9.5  0.0 18.6

 83.9   0.5866 0.1411 0.1470   21.7   9.2 13.0 10.7  9.6  2.3
                               10.5  17.1  7.9 13.1  8.3  0.0

 ----------------------------------------------------------------

 41.5   0.8460 0.3460 0.1540    0.0  28.2 29.5 28.4 29.4 27.5 25.8
                               84.8  10.6 12.6  0.0  2.2 21.0  0.0

 14.1   0.7500 0.3606 0.0000    0.0  27.0 27.8 27.0 29.4 23.5 24.2 14.2
                                0.0   0.0  0.0  0.0  0.0  0.0  0.0 23.7

 PATFOM =  11.91
```

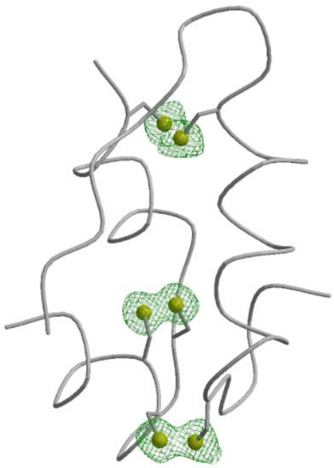

Figure S3 Anomalous map from *SHELXE* contoured at 3σ in insulin contoured around the disulfide sulfurs

S3.1.5. Density Modification and Autotracing in *SHELXE*

Global autotracing cycle 3

```
<cos> 0.665 / 0.482  <fom> 0.300 / 0.280  MPE  39.0 / 46.6  wMPE 34.4 / 34.9
<wt> = 0.299, Contrast = 0.547, Connect. = 0.808 for dens.mod. cycle 1
<cos> 0.726 / 0.642  <fom> 0.300 / 0.289  MPE  33.7 / 32.3  wMPE 28.3 / 23.9
<wt> = 0.299, Contrast = 0.713, Connect. = 0.849 for dens.mod. cycle 2
<cos> 0.754 / 0.666  <fom> 0.300 / 0.291  MPE  31.2 / 30.1  wMPE 26.2 / 21.6
<wt> = 0.299, Contrast = 0.948, Connect. = 0.883 for dens.mod. cycle 3
<cos> 0.769 / 0.682  <fom> 0.300 / 0.293  MPE  29.8 / 28.6  wMPE 25.2 / 21.0
<wt> = 0.299, Contrast = 0.969, Connect. = 0.887 for dens.mod. cycle 4
<cos> 0.777 / 0.703  <fom> 0.300 / 0.292  MPE  29.1 / 26.8  wMPE 24.7 / 19.6
<wt> = 0.299, Contrast = 0.993, Connect. = 0.890 for dens.mod. cycle 5
<cos> 0.776 / 0.682  <fom> 0.300 / 0.293  MPE  29.1 / 28.6  wMPE 24.5 / 19.8
<wt> = 0.299, Contrast = 0.998, Connect. = 0.891 for dens.mod. cycle 6
<cos> 0.779 / 0.705  <fom> 0.300 / 0.292  MPE  28.8 / 26.6  wMPE 24.4 / 18.9
<wt> = 0.299, Contrast = 1.005, Connect. = 0.892 for dens.mod. cycle 7
<cos> 0.778 / 0.692  <fom> 0.300 / 0.292  MPE  28.9 / 27.7  wMPE 24.5 / 19.3
<wt> = 0.299, Contrast = 1.008, Connect. = 0.892 for dens.mod. cycle 8
<cos> 0.778 / 0.709  <fom> 0.300 / 0.292  MPE  28.9 / 26.2  wMPE 24.6 / 18.7
<wt> = 0.299, Contrast = 1.009, Connect. = 0.892 for dens.mod. cycle 9
<cos> 0.778 / 0.711  <fom> 0.300 / 0.292  MPE  28.9 / 26.0  wMPE 24.7 / 19.2
<wt> = 0.299, Contrast = 1.011, Connect. = 0.892 for dens.mod. cycle 10

Shift from model in .ent:  dx=-0.000  dy=-0.000  dz=-0.000

NOGO map generated for regions about rotation axes (if any)
  6 heavy atoms with Occ*Z >  4.80 added to NOGO map

  407 peaks > 0.5 sigma used to seed fragment search
Space for about   78 unique residues taking solvent into account

   56 potential tripeptides employed

Using tripeptides from previous cycle as seeds

  0 Splices to join chains,  0 cis peptide(s) found

Global chain diagnostics:
 4.55 40.91 26.26 13.64  2.53  2.02  2.53  2.02  0.00   5.556  77.495   0.357
 92.2% of CA within 1.0A,  90.2% within 0.5A and   5.9% incorrect CA

   49 residues left after pruning, divided into chains as follows:
A:  23   B:  26
 4.55 40.91 26.26 13.64  2.53  2.02  2.53  2.02  0.00   5.556  77.495   0.357
 94.1% of correct side chains,   0.0% incorrect

CC for partial structure against native data =  58.23 %

Phases from trace:
<cos> 0.637 / 0.558  <fom> 0.839 / 0.916  MPE  41.2 / 39.8  wMPE 37.1 / 35.5

Combined phases:
<cos> 0.684 / 0.572  <fom> 0.901 / 0.893  MPE  37.1 / 38.5  wMPE 32.7 / 31.2
```

S3.1.6. R_{free} Reflections

Generation of an R_{free} set starting form one randomly selected reflection: 3 1 46. There are 7 reflections in the HKLF5 data set with contribution of this reflection:

```
  3   1  46 42.4446 3.68425  -2
 24  28  28 42.4446 3.68425   1
  3   1  46 26.9011 3.10304  -2
 28  19  31 26.9011 3.10304   1
  3   1  46 14.4297 7.03810  -2
```

```
28   0  36 14.4297 7.03810   1
 3   1  46 5.76621 1.37292   2
 3   1  46 9.79115 2.20466   1
25  27  28 18.0364 7.71839  -2
 3   1  46 18.0364 7.71839   1
16  24  36 9.21336 6.79040  -2
 3   1  46 9.21336 6.79040   1
```

The reflection 24 28 28 has the following contributions in the HKLF5 data set:

```
24  28  28 29.8134 1.67879   1
15  24  37 53.3834 5.64720  -2
24  28  28 53.3834 5.64720   1
24  28  28 29.9736 3.99235   2
 3   1  46 42.4446 3.68425  -2
24  28  28 42.4446 3.68425   1
18  20  38 39.1706 7.55775  -2
24  28  28 39.1706 7.55775   1
24  28  28 24.6660 5.09521  -2
28  11  35 24.6660 5.09521   1
24  28  28 19.7911 7.94195  -2
18  21  37 19.7911 7.94195   1
24  28  28 45.6375 8.32085  -2
16  16  40 45.6375 8.32085   1
```

The other reflections that overlap with 3 1 46 again have several new overlaps. Therefore, adding step by step all twin-related reflections leads to ca. 90 % of the data starting with just one reflection

Table S4 Number of R_{free} reflections starting from reflection 3 1 46

dataset	domain 1	domain 2	domain 1-2
data	11560	11531	11663
R_{free} reflections	10417	10417	10424
percentage	90.1	90.3	89.3

Table S5 Distribution of reflections that are not twin-related to those of Table S4

Resolution	#Data	#Theory	%Complete
Inf -15.30	19	20	95
15.30 - 9.46	39	47	83
9.46 - 7.30	55	67	82.1
7.30 - 5.43	59	176	33.5
5.43 - 4.17	54	341	15.8
4.17 - 3.59	57	347	16.4
3.59 - 3.09	56	534	10.5
3.09 - 2.74	58	661	8.8
2.74 - 2.39	56	1060	5.3
2.39 - 2.12	56	1377	4.1
2.12 - 1.80	57	2867	2
1.80 - 1.59	81	3319	2.4
1.59 - 1.58	50	189	26.5
1.58 - 1.57	87	211	41.2
1.57 - 1.56	118	191	61.8
1.56 - 1.55	171	261	65.5
1.55 - 1.54	68	196	34.7

S3.2. Glucose Isomerase

S3.2.1. Cell Determination in *CELL_NOW*

```
   4427 reflections read from file: gdri2_0m.p4p

 Searching for vectors with 90.00 < d < 110.00, superlattice threshold = 10.0%

The following cells would appear to be plausible, but should be checked using
 XPREP because they are not necessarily the conventional cells.

 FOM, % within 0.2, a..gamma, volume and lattice type for potential unit-cells:

   1 1.000  42.3   92.539  98.848 103.610   90.29   90.18   90.68  947659.3  I

 ------------------------------------------------------------------------------

 Cell for domain  1:   92.539   98.848  103.610    90.29    90.18    90.68

 Figure of merit: 0.359   %(0.1):  34.1   %(0.2):  43.9   %(0.3):  53.8

 Orientation matrix:  0.00593817 -0.00837226 -0.00048952
                     -0.00899448 -0.00557699 -0.00120486
                      0.00079449  0.00107888 -0.00956378

 Percentages of reflections in this domain not consistent with lattice types:
 A: 50.2,  B: 51.0,  C: 50.8,  I:  7.4,  F: 76.0,  O: 65.6  and  R: 66.4%

 Percentages of reflections in this domain that do not have:
 h=2n: 50.6,  k=2n: 51.0,  l=2n: 51.4,  h=3n: 67.0,  k=3n: 67.2,  l=3n: 66.1%


  2145 reflections within tolerance assigned to domain  1,
  2145 of them exclusively;  2282 reflections not yet assigned to a domain

 ------------------------------------------------------------------------------

 Cell for domain  2:   92.539   98.848  103.610    90.29    90.18    90.68

 Figure of merit: 0.293   %(0.1):  15.7   %(0.2):  48.6   %(0.3):  63.1

 Orientation matrix: -0.00046763 -0.00378916  0.00892166
                     -0.00849873 -0.00575178 -0.00258504
                      0.00665933 -0.00741086 -0.00262273

 Rotated from first domain by  76.5 degrees about
 reciprocal axis  1.000  0.885  0.238  and real axis  1.000  0.780  0.194

 Twin law to convert hkl from first to      0.671   0.497  -0.465
 this domain (SHELXL TWIN matrix):          0.216   0.542   0.783
                                            0.792  -0.682   0.253

  1691 reflections within tolerance assigned to domain  2,
  1302 of them exclusively;   980 reflections not yet assigned to a domain

 ------------------------------------------------------------------------------

 Cell for domain  3:   92.539   98.848  103.610    90.29    90.18    90.68

 Figure of merit: 0.339   %(0.1):  27.0   %(0.2):  82.7   %(0.3):  91.9

 Orientation matrix: -0.00597407  0.00218951  0.00774348
                     -0.00893795 -0.00039094 -0.00544524
                     -0.00110330 -0.00986989  0.00188285

 Rotated from first domain by 122.2 degrees about
```

```
reciprocal axis  1.000  0.572  0.713  and real axis  1.000  0.511  0.569

Twin law to convert hkl from first to      0.373   0.849   0.198
this domain (SHELXL TWIN matrix):          0.075  -0.271   0.915
                                           1.034  -0.350  -0.169

 1495 reflections within tolerance assigned to domain  3,
  873 of them exclusively;   107 reflections not yet assigned to a domain
```

S3.2.2. Excerpt of the *TWINABS* output for Glucose Isomerase

```
  237474 data (  47082 unique ) involve domain  1 only,  mean I/sigma   9.0
  237699 data (  51266 unique ) involve domain  2 only,  mean I/sigma   8.2
  237893 data (  48298 unique ) involve domain  3 only,  mean I/sigma   5.7
  126763 data (  75981 unique ) involve   2 domains,  mean I/sigma  10.4
   10261 data (   7435 unique ) involve   3 domains,  mean I/sigma   9.9


Unique HKLF 4 data extracted from all observed data

 Cycle   N(1) Rint(1)  N(all) Rint(all) Twin fractions
    1  316840  0.0522  776204  0.0663   0.4363  0.4055  0.1582
    2  332092  0.0548  847949  0.0593   0.4359  0.4060  0.1581
    3  332094  0.0547  847951  0.0592   0.4359  0.4060  0.1581
    4  332094  0.0546  847951  0.0592   0.4359  0.4060  0.1581
    5  332094  0.0546  847951  0.0592   0.4359  0.4060  0.1580
    6  332094  0.0546  847951  0.0592   0.4359  0.4060  0.1580
    7  332094  0.0546  847951  0.0592   0.4359  0.4060  0.1580
    8  332094  0.0546  847951  0.0592   0.4359  0.4060  0.1580
    9  332094  0.0546  847951  0.0592   0.4359  0.4060  0.1580
   10  332094  0.0546  847951  0.0592   0.4359  0.4060  0.1580
   11  332094  0.0546  847951  0.0592   0.4359  0.4060  0.1580
   12  332094  0.0546  847951  0.0592   0.4359  0.4060  0.1580
   13  332094  0.0546  847951  0.0592   0.4359  0.4060  0.1580
   14  332094  0.0546  847951  0.0592   0.4359  0.4060  0.1580
   15  332094  0.0546  847951  0.0592   0.4359  0.4060  0.1580
   16  332094  0.0546  847951  0.0592   0.4359  0.4060  0.1580
   17  332094  0.0546  847951  0.0592   0.4359  0.4060  0.1580
   18  332094  0.0546  847951  0.0592   0.4359  0.4060  0.1580
   19  332094  0.0546  847951  0.0592   0.4359  0.4060  0.1580
   20  332094  0.0546  847951  0.0592   0.4359  0.4060  0.1580

 N(1) and Rint(1) refer to singles and composites that include domain 1.

 Rint = 0.0592  for all  847951  observations and
 Rint = 0.0508  for all  531048  observations with I > 3sigma(I)
```

S3.2.3. Excerpt of the *XPREP* output for Glucose Isomerase

```
SPACE GROUP DETERMINATION

Lattice exceptions:  P      A      B      C      I      F     Obv    Rev    All

N (total) =          0  62821  62830  62819      0  94235  83880  83791 125668
N (int>3sigma) =     0  43885  44003  43966      0  65927  58456  58407  87790
Mean intensity =   0.0    4.8    4.8    4.8    0.0    4.8    4.8    4.8    4.8
Mean int/sigma =   0.0   18.5   18.5   18.4    0.0   18.5   18.4   18.5   18.4

Crystal system O and Lattice type I selected

Mean |E*E-1| = 0.768 [expected .968 centrosym and .736 non-centrosym]

Systematic absence exceptions:

         b--   c--   -c-   -a-   --a   --b

N        793   793   759   759   724   724
N I>3s  510   510   541   541   510   510
<I>      8.0   8.0   8.1   8.1   8.5   8.5
<I/s>  24.8  24.8  25.9  25.9  23.6  23.6

Identical indices and Friedel opposites combined before calculating R(sym)

Option  Space Group  No.  Type  Axes  CSD  R(sym) N(eq)  Syst. Abs.   CFOM

[A] I222           # 23  chiral   1    17  0.000      0  0.0 / 18.4   6.20
[B] I2(1)2(1)2(1)  # 24  chiral   1     9  0.000      0  0.0 / 18.4  10.65
[C] Imm2           # 44  non-cen  1     8  0.000      0  0.0 / 18.4  11.76
[D] Imm2           # 44  non-cen  5     8  0.000      0  0.0 / 18.4  11.76
[E] Imm2           # 44  non-cen  3     8  0.000      0  0.0 / 18.4  11.76
[F] Immm           # 71  centro   1     7  0.000      0  0.0 / 18.4  17.04

Estimation of SAD delta-F values

Local scaling employed with radius of 0.1847 reciprocal Angstroms

 Anomalous signal/noise ratios (1.0 is random).  The first line is based on

 input sigmas, the second on variances of F+ and F- (if not already averaged):

 Inf - 8.0 - 6.0 - 5.0 - 4.0 - 3.5 - 3.0 - 2.6 - 2.4 - 2.2 - 2.0 - 1.8 - 1.6 A

    3.46  3.76  3.67  2.86  2.19  1.69  1.68  1.72  1.58  1.41  1.28  1.12

    61.3  Neighbors used on average for F+/F- local scaling
 Ranom =    0.0660  before and   0.0661  after local scaling
```

S3.2.4. Substructure Solution in *SHELXD*

```
PSUM 70.58  PSMF Peaks: 156 97 23 22 21 21 20 20 19 19 18 18
 Cycle 12  Peaks 99 53 20 20 19 18
 R = 0.363, Min.fun. = 0.472, <cos> = 0.497, Ra = 0.358

    x       y       z      sof  height
 0.91828 0.63165 0.06579  1.000  99.90
 0.86971 0.64425 0.08481  1.000  53.89
 0.88692 0.67207 0.24079  1.000  21.05
 0.79707 0.68810 0.10560  1.000  20.52
 0.93193 0.64911 0.18688  1.000  19.31
 0.83399 0.74237 0.23487  1.000  18.23

 Minimum distances (top row, 0 if special position) and PSMF (bottom row)

 Peak    x      y      z      self  cross-vectors

 99.9   0.9183 0.6316 0.0658   20.3
                              115.6

 53.9   0.8697 0.6442 0.0848   29.8   5.1
                               34.1  58.5

 21.1   0.8869 0.6721 0.2408   39.7  18.6 16.3
                                0.0  16.8  6.7

 20.5   0.7971 0.6881 0.1056   42.8  13.2  8.3 16.3
                                0.0  17.7  0.7  0.7

 ------------------------------------------------------

 19.3   0.9319 0.6491 0.1869   31.8  12.6 12.0  7.3 15.5
                                0.8  17.2  8.0  0.0  0.3

 18.2   0.8340 0.7424 0.2349   46.6  21.9 18.5  8.5 14.7 13.8
                                9.9  19.1  5.2  0.0  0.0  0.0

 PATFOM =  25.10
```

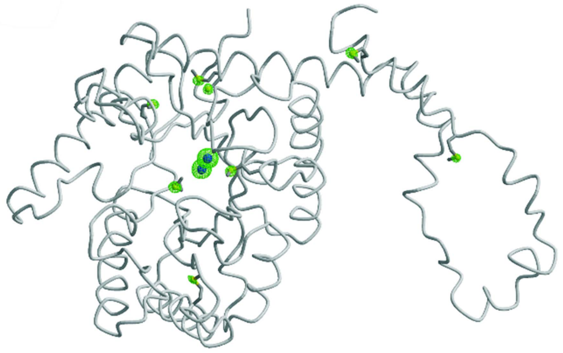

Figure S4 Anomalous map from *SHELXE* contoured at 3σ in glucose isomerease, contoured around the 1.5 Mn atoms and some Methionine sulfurs

S3.2.5. Density Modification and Autotracing in *SHELXE*

```
Global autotracing cycle   3

 <cos> 0.619 / 0.480  <fom> 0.300 / 0.289  MPE  42.9 / 46.8  wMPE 37.0 / 34.5
 <wt> = 0.294, Contrast = 0.422, Connect. = 0.722 for dens.mod. cycle 1
 <cos> 0.674 / 0.627  <fom> 0.300 / 0.312  MPE  38.3 / 33.6  wMPE 31.2 / 24.5
 <wt> = 0.294, Contrast = 0.526, Connect. = 0.760 for dens.mod. cycle 2
 <cos> 0.701 / 0.627  <fom> 0.300 / 0.320  MPE  36.0 / 33.6  wMPE 28.9 / 23.1
 <wt> = 0.294, Contrast = 0.658, Connect. = 0.800 for dens.mod. cycle 3
 <cos> 0.720 / 0.648  <fom> 0.300 / 0.320  MPE  34.4 / 31.7  wMPE 27.6 / 21.8
 <wt> = 0.294, Contrast = 0.674, Connect. = 0.805 for dens.mod. cycle 4
 <cos> 0.730 / 0.655  <fom> 0.300 / 0.319  MPE  33.5 / 31.0  wMPE 27.1 / 21.6
 <wt> = 0.294, Contrast = 0.690, Connect. = 0.810 for dens.mod. cycle 5
 <cos> 0.736 / 0.657  <fom> 0.300 / 0.318  MPE  33.0 / 30.8  wMPE 26.7 / 21.5
 <wt> = 0.294, Contrast = 0.696, Connect. = 0.812 for dens.mod. cycle 6
 <cos> 0.740 / 0.655  <fom> 0.300 / 0.316  MPE  32.7 / 31.1  wMPE 26.6 / 21.2
 <wt> = 0.294, Contrast = 0.703, Connect. = 0.814 for dens.mod. cycle 7
 <cos> 0.742 / 0.661  <fom> 0.300 / 0.316  MPE  32.5 / 30.6  wMPE 26.6 / 21.3
 <wt> = 0.294, Contrast = 0.705, Connect. = 0.815 for dens.mod. cycle 8
 <cos> 0.742 / 0.663  <fom> 0.300 / 0.316  MPE  32.5 / 30.3  wMPE 26.6 / 21.3
 <wt> = 0.294, Contrast = 0.708, Connect. = 0.816 for dens.mod. cycle 9
 <cos> 0.742 / 0.668  <fom> 0.300 / 0.316  MPE  32.5 / 29.8  wMPE 26.7 / 21.3
 <wt> = 0.294, Contrast = 0.708, Connect. = 0.816 for dens.mod. cycle 10

 Shift from model in .ent:  dx=-0.000  dy=-0.500  dz=-0.000

 NOGO map generated for regions about rotation axes (if any)
   2 heavy atoms with Occ*Z >  7.50 added to NOGO map

  3185 peaks > 0.5 sigma used to seed fragment search
 Space for about  475 unique residues taking solvent into account

   405 potential tripeptides employed


 Using tripeptides from previous cycle as seeds
 Z:    6   3.915   1.585   0.584   0.498   0.800   0.400   N 0.873
  4.00 12.00 20.00 12.00  0.00  4.00  4.00  0.00  0.00  44.000   1.296   1.016

   0 Splices to join chains,  0 cis peptide(s) found

 Global chain diagnostics:
  3.31 18.90 29.20 25.11 11.85  5.15  1.69  0.92  0.56   3.315  73.529   0.358
  90.5% of CA within 1.0A,  80.9% within 0.5A and   1.8% incorrect CA

   352 residues left after pruning, divided into chains as follows:
 A:  15   B:  17   C:  37   D:   8   E:  64   F:  51   G:   6   H:  34   I:  75
 J:  45
  3.31 18.83 29.27 25.11 11.85  5.08  1.76  0.92  0.56   3.315  73.529   0.358
  85.8% of correct side chains,   5.7% incorrect

 CC for partial structure against native data =  50.04 %

 Phases from trace:
 <cos> 0.581 / 0.523  <fom> 0.796 / 0.899  MPE  45.8 / 42.9  wMPE 40.7 / 38.6

 Combined phases:
 <cos> 0.644 / 0.536  <fom> 0.869 / 0.876  MPE  40.7 / 41.7  wMPE 35.4 / 33.8
```

This full sidechain tracing is in an early stage of development and is likely to get better.

S3.2.6. R_{free} reflections

Table S6 Number of R_{free} reflections starting from reflection 9 35 16

dataset	domain 1	domain 2	domain 3	domain 1-2	domain 1-3
unique data	51907	57458	52886	61320	61832
R_{free} reflections	42268	46315	43416	56305	56764
percentage	81.4	80.6	82.1	91.8	91.8

Table S7 Distribution of reflections that are not twin-related to those of Table S6

Resolution	#Data	#Theory	%Complete
Inf -16.75	73	73	100
16.75 -10.83	170	175	97.1
10.83 - 8.03	243	323	75.2
8.03 - 5.75	243	899	27
5.75 - 3.88	244	3106	7.9
3.88 - 3.13	244	3995	6.1
3.13 - 2.57	240	6702	3.6
2.57 - 2.22	267	8259	3.2
2.22 - 2.15	233	2358	9.9
2.15 - 2.08	235	2635	8.9
2.08 - 2.00	260	3503	7.4
2.00 - 1.94	244	3018	8.1
1.94 - 1.88	255	3405	7.5
1.88 - 1.83	222	3192	7
1.83 - 1.79	231	2828	8.2
1.79 - 1.74	254	3881	6.5
1.74 - 1.70	253	3445	7.3
1.70 - 1.67	222	2804	7.9
1.67 - 1.64	269	3024	8.9
1.64 - 1.61	268	3211	8.3
1.61 - 1.60	200	1126	17.8

References

Sparks, R. A. (2000). *GEMINI*. Madison, WI, USA.
